# Forms of Non-Apoptotic Cell Death and Their Role in Gliomas—Presentation of the Current State of Knowledge

**DOI:** 10.3390/biomedicines12071546

**Published:** 2024-07-11

**Authors:** Reinhold Nafe, Elke Hattingen

**Affiliations:** Department of Neuroradiology, Clinics of Johann Wolfgang Goethe-University, Schleusenweg 2-16, D-60528 Frankfurt am Main, Germany; hattingen@med.uni-frankfurt.de

**Keywords:** programmed cell death, gliomas, cancer, tumor cells, tumor environment, autophagy, ferroptosis, pyroptosis, necroptosis, cuproptosis

## Abstract

In addition to necrosis and apoptosis, the two forms of cell death that have been known for many decades, other non-apoptotic forms of cell death have been discovered, many of which also play a role in tumors. Starting with the description of autophagy more than 60 years ago, newer forms of cell death have become important for the biology of tumors, such as ferroptosis, pyroptosis, necroptosis, and paraptosis. In this review, all non-apoptotic and oncologically relevant forms of programmed cell death are presented, starting with their first descriptions, their molecular characteristics, and their role and their interactions in cell physiology and pathophysiology. Based on these descriptions, the current state of knowledge about their alterations and their role in gliomas will be presented. In addition, current efforts to therapeutically influence the molecular components of these forms of cell death will be discussed. Although research into their exact role in gliomas is still at a rather early stage, our review clarifies that all these non-apoptotic forms of cell death show significant alterations in gliomas and that important insight into understanding them has already been gained.

## 1. Introduction

A comprehensive description of non-apoptotic forms of programmed cell death (PCD) in certain tumor types should include a detailed presentation of all oncologically relevant forms, namely ferroptosis, pyroptosis, necroptosis, cuproptosis, paraptosis, methuosis, parthanatos, and anoikis. Compared to the intensively researched autophagy, autophagic cell death has been researched less extensively to date and can therefore be mentioned in the context of the new forms of PCD. The overall presentation will show that the significance of the individual forms of PCD for tumor biology and thus for the promotion or inhibition of tumor growth can be different for the various tumor types, and that separate research into PCD is therefore important for individual tumor types such as gliomas. It is also necessary to discuss those forms of PCD that have not yet been investigated in gliomas, or only to a limited extent, but which could become increasingly important in this type of tumor in the future. Finally, the current approaches to molecularly influence the signaling pathways of PCDs in gliomas are discussed, which represent potential therapeutic strategies for the treatment of patients with gliomas.

## 2. Autophagy

**Figure 1:**
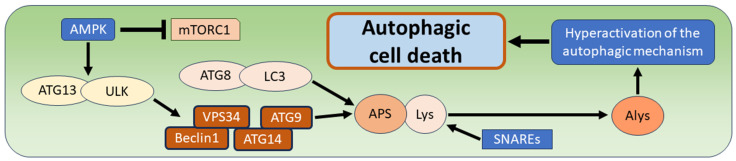
The central signaling pathways of autophagic cell death: AMP-activated protein kinase (AMPK) promotes the initiation of autophagy by blocking the inhibitory mammalian target of rapamycin complex 1 (mTORC1) and by activating the Unc-51-like kinase complex (ULK) and several autophagy related proteins (ATGs) such as ATG13; ULK leads to the recruitment of the phosphatidylinositol 3-kinase complex with type 3 catalytic subunit (PIK3C3 complex), which consists of vacuolar protein sorting 34 (VPS34) and several ATGs such as Beclin1 (ATG6), ATG9, and ATG14; incorporation of the ubiquitin-like conjugation system, including ATG8 and lipidated light chain 3 (LC3), into the autophagosomal membrane leads to fusion of the autophagosome (APS) with lysosomes (Lys) to form an autolysosome (Alys); this fusion is supported by soluble N-ethylmaleimide-sensitive-factor-attachment protein receptors (SNAREs); hyperactivation of this autophagic mechanism leads to autophagic cell death [8,9,10].

The term “autophagy” refers to a process of cellular self-preservation in which cells achieve the degradation of their own components under physiological conditions. The first observations were made in 1956 by Clarke in the proximal tubules of newborn mice, as well as in 1962 by Ashford and Porter in liver cells. In both studies, large vacuoles with amorphous material and dense inclusions were observed, which was interpreted as a physiological autolytic process confined to the vacuoles and separated from other cellular compartments by membranes [1,2]. A few years later, the term “autophagy” was used in various studies, with the common observation of autophagic vacuoles stained with acid phosphatase suggesting that lysosomal enzymes are the main players in this digestive process [3,4,5,6]. In the context of diseases or especially tumors, the term “autophagy” has been used more broadly and refers to the occurrence of this phenomenon under pathological conditions, whereby autophagy can promote or even prevent the further progression of the disease. Although double-membrane autophagic vacuoles, referred to as “autophagosomes”, are considered a typical morphological feature of autophagy, the definition of autophagy is based on the detection of activated specific proteins encoded by autophagy-related genes (“ATGs”) and additional genes known to be involved in the process of autophagy [7]. In addition to this type of autophagy, which is also referred to as “macro-autophagy”, two other types of autophagy have been described, namely “micro-autophagy” and “chaperone-mediated autophagy”. Micro-autophagy leads to the sequestration of KFERQ-like motif-bearing proteins by direct membranous invaginations formed on the surface of lysosomes. Chaperone-mediated autophagy leads to lysosomal degradation of the KFERQ-like motif-bearing proteins that are first recognized by the heat shock protein family A member 6 (HSPA8) [8]. The molecular cascade of macro-autophagy is extensive and begins with the activation of the Unc-51-like kinase complex (“ULK”) and several autophagy related proteins such as ATG13 by the AMP-activated protein kinase (AMPK), which also inhibits the mammalian target of rapamycin complex 1 (mTORC1). ULK leads to the recruitment of the phosphatidylinositol 3-kinase complex with type 3 catalytic subunit (“PIK3C3”), which contains several pro-autophagic factors such as vacuolar protein sorting 34 (“VPS34”), Beclin1 (ATG6), ATG9, and ATG14. Another important step is the incorporation of the ubiquitin-like conjugation system ATG8/LC3, including the lipidated ATG8 and the lipidated light chain 3 (LC3), into the autophagosomal membrane, followed by the fusion of the autophagosome with lysosomes to form an autolysosome in which the degradation of autophagic substrates by acidic lysosomal hydrolases takes place. This fusion of autophagosomes with lysosomes is mediated by soluble N-ethylmaleimide-sensitive-factor-attachment protein receptors (SNAREs) [8,9,10]. In contrast to the well-described molecular cascade of autophagy, the phenomenon of autophagic cell death is poorly understood. The general assumption is that hyperactivation of the autophagic mechanism promotes cell death. It is recommended to use the term “autophagic cell death” only when there is clear evidence of a link between the cell death phenomenon and the activity of the molecular autophagy cascade (Figure 1) (Table 1) [8,9].

Studies on autophagy in cancer have shown that the alterations in the molecular cascade are not necessarily comparable between different tumor types. It should also be noted that both the significance of autophagy itself and the special case of autophagic cell death for the individual tumor entity must be investigated separately [8,10]. Autophagy was confirmed in both high-grade and low-grade gliomas by the presence of the three autophagy-associated proteins, namely light-chain 3 (LC3), Beclin1, and p62. The expression of these three proteins were observed to occur more frequently in high-grade gliomas than in low-grade gliomas, and the positivity of at least two of these three proteins was significantly associated with a worse prognosis of patients [43]. In another study, the ratio between LC3 and p62 was interpreted as a measure of autophagic activity, with the ratio being higher in high-grade gliomas [44]. Experimental blockade of autophagy-related proteins in gliomas confirmed the tumor-promoting properties of autophagy [45] and its contribution to the maintenance of glioma stem cells [44,46]. A particular problem would be the temozolomide (TMZ) resistance of gliomas due to the induction of cytoprotective autophagy by TEM. Experimental blockade of dipeptidyl peptidase 4 (DPP4), otherwise known as a stem cell marker, led to an inhibition of the autophagic Beclin1 pathway and to an enhancement of TMZ-induced cytotoxicity in glioma cells. Therefore, the DPP4 inhibitor, sitagliptin, used in this study is considered a potential therapeutic option for glioma patients to overcome TMZ resistance [47]. In contrast to these tumor-promoting properties of autophagy reported in several studies, autophagic cell death shows the opposite effect due to its anti-tumor efficacy, as confirmed in other studies. Experimental inhibition of the SHH pathway with the SHH antagonist LDE225 and the interfering RNA molecules applied with a lentivirus resulted in autophagic cell death of glioma stem cells expressing the stem cell marker CD133 [48]. Other in vitro observations also confirmed the induction of autophagic cell death in glioma cells by application of the opioid receptor agonist, loperamide, the dopamine receptor antagonist, pimozide, and the antipsychotic, phenothiazine [49,50]. For loperamide and pimozide, it was postulated that autophagic cell death occurred via excessive accumulation of lysosomal sphingolipids and subsequent lysosomal dysfunction [49]. For phenothiazine, the authors emphasized the need for further investigation of the signaling pathways involved [50,51]. In addition to the tumor cells themselves, the process of autophagy can also influence the cellular components of the tumor environment. A groundbreaking observation was made in the exosomes released from glioma cells, which favored autophagy and led to an immunosuppressive phenotype of macrophages that promoted proliferation and migration of gliomas in vitro and in vivo. In vitro analyses support the assumption that interleukin 6 (Il-6) contained in the exosomes and the micro-RNA 155-3p support autophagy and the associated tumor-promoting phenotype of macrophages [52]. Experimental inhibition of autophagy in myeloid-derived suppressor cells (MDSCs) in melanomas led to the efficient activation of tumor-specific CD4 T cells, impaired immunosuppressive function of MDSCs, and a significant reduction in tumor volume [53]. Impaired growth of mammalian, prostate, and colorectal tumors was detected in mice lacking ATG genes 5, 14, and 16L1. In addition, ATG5-deficient CD8 T cells showed a marked shift towards IFN-γ and tumor necrosis factor α (TNF-α), resulting in the effector memory cell phenotype. Consistent with this, the adoptive transfer of ATG5-deficient CD8 T cells resulted in improved tumor control [54]. Clear confirmation of the association of autophagy and prognosis of patients with gliomas was achieved by gene signature analysis of data from over 1500 patients and over 1000 samples from a validation cohort. Several prognostically relevant, differentially expressed autophagy-related genes (ATG genes) were identified, which represent a statistically independent prognostic factor. Notably, a significant positive correlation between this group of prognostically relevant ATG genes and the expression pattern of proteins related to an immunosuppressive tumor environment, such as programmed cell death ligand (PD-L1), was confirmed [55]. The current results support the goal of therapeutically targeting appropriate molecular components of tumor-protective autophagy in gliomas, but there is a consensus that further studies are needed to elucidate the overall interrelationship between autophagy and the tumor environment in gliomas and in cancer in general [55,56].

The molecular interaction between the various forms of cell death is very extensive and has so far only been incompletely understood. Recent findings on autophagy indicate that it has a molecular interaction with apoptosis and particularly ferroptosis. A common player in the initiation of autophagy and apoptosis is the tumor protein 53 (TP53), whose activity includes both the maturation of the autophagosome and the regulation of apoptosis through activation or suppression of proteins involved in the apoptotic process [57]. Oxidative stress caused by an excess of reactive oxygen species (ROS) is another factor that can trigger apoptotic and autophagic cell death programs. Mitochondria are considered the main source of ROS in autophagy signaling, but other signaling pathways such as the mitogen-activated protein kinase pathway (MAPK) or the mammalian target of rapamycin pathway (mTOR) are also involved in the induction of autophagy by ROS. Both autophagy inducing cell death and protective autophagy can be achieved, as well as the simultaneous initiation of autophagy and apoptosis [58,59]. The wingless-type signaling pathway (WNT) can act as a regulatory network that promotes or inhibits apoptosis and autophagy, including the activation or suppression of caspases or ATG proteins [60]. Proteins of the autophagy cascade can promote ferroptosis, such as Beclin1, by binding to the solute carrier family 7 member 11 (SLC7A11) and leading to the blockade of the cystine/glutamate antiporter system Xc-, which is the central target of the molecular ferroptosis cascade [61]. As another important link between autophagy and ferroptosis, transmembrane protein 164 (TMEM164), which targets autophagy-related protein 5 (ATG5), has been shown to contribute to the degradation of ferritin and glutathione peroxidase 4 (GPX4), leading to increased iron accumulation and peroxidation with triggering ferroptosis [62]. Experimental silencing of certain proteins that are upregulated in tumors can also lead to a simultaneous initiation of autophagy and ferroptosis. This was confirmed for the coatamer–protein complex subunit zeta 1 (COPZ1), which is upregulated in gliomas and is involved in autophagy regulation and iron metabolism. Knockdown of COPZ1 in a glioblastoma cell line led to the induction of autophagy and degradation of ferritin with subsequent ferroptosis. Therefore, COPZ1 is considered a new therapeutic target for the treatment of human glioblastomas [63].

## 3. Ferroptosis

**Figure 2:**
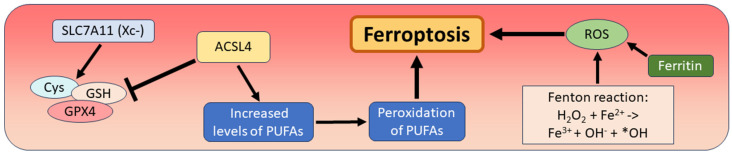
The central signaling pathways of ferroptosis: Acyl-CoA synthetase long-chain family member 4 (ACSL4) is the main promoter of ferroptosis, leading to blockade of the cellular antioxidant system consisting of cysteine (Cys), glutathione (GSH), and glutathione peroxidase 4 (GPX4), which is activated by the cysteine/glutamate antiporter system Xc- with its main component solute carrier family 7 member 11 (SLC7A11); ACSL4 also increases the amount of polyunsaturated fatty acids (PUFAs), and the subsequent peroxidation of PUFAs promotes ferroptosis, as well as the increased production of reactive oxygen species (ROS) catalyzed by intracellular ferritin and by the Fenton reaction [12,65,66].

In the initial description by Dixon and coworkers, a new type of cell death was described as “a unique iron-dependent form of non-apoptotic cell death”, which was termed “ferroptosis”. A trigger function for ferroptosis was discovered for erastin, which acts as an inhibitor of the cysteine/glutamate antiporter system Xc- with its main component solute carrier family 7 member 11 (SLC7A11), leading to an inhibition of the antioxidant defense mechanisms of the tumor cells and cell death of several RAS-mutated tumor cell lines [11]. The molecular cascade of ferroptosis has been described in many subsequent studies, with the common observation that the antioxidant system against ferroptosis, including cysteine (Cys), glutathione (GSH), and glutathione peroxidase 4 (GPX4), can be blocked by various compounds. One of the key compounds that promotes ferroptosis is acyl-CoA synthetase long-chain family member 4 (ACSL4), an enzyme involved in fatty acid metabolism and increasing levels of polyunsaturated fatty acids (PUFAs). Peroxidation reactions of these PUFAs lead to subsequent disruption of cell membranes and organelle membranes [64,65,66]. Another effect of the suppression of the cellular antioxidant system is the so-called “Fenton reaction”, which leads to the formation of hydroxides and reactive oxygen species (ROS) through the reaction between Fe^2+^ ions and hydrogen peroxide and triggers ferroptosis. In this reaction, ferritin represents a further trigger for the formation of ROS and thus for ferroptotic cell death (Figure 2) [12].

In addition to the Xc/GSH/GPX4 axis mentioned above, another important inhibitor of ferroptosis is the “ferroptosis suppressor protein 1” (FSP1), which promotes the formation of the antioxidant ubiquinol via the FSP1–coenzyme Q10 (CoQ10)–NAD(P)H axis. FSP1 is associated with poor prognosis in malignant tumors and, together with GPX4, is considered an important target for cancer treatment [66]. Glioma cells have a high requirement for iron, and the key molecular systems and their alterations leading to ferroptosis have been confirmed in several experimental and in vitro studies as follows: The level of the most important iron storage protein, ferritin, is elevated in the serum, cerebrospinal fluid, and resected glioma tissue of patients with gliomas [67,68]. A small part of the iron is not stored in the ferritin complex but is present in the cells and tends to catalyze the formation of ROS [69]. Transferrin 2 and its receptor are highly expressed in glioma cells and promote intracellular transport of iron, production of ROS, and lipid peroxidation, thereby promoting ferroptosis [70]. However, it should be emphasized that the exact mechanisms and the relationship between the dysregulation of iron metabolism and cancer progression are still largely unclear [67]. Glioma cells show a high expression level of GPX4, which leads to tumor progression and chemoresistance. The solute carrier SLC7A11 is also upregulated, promoting the uptake of cysteine and supporting GSH synthesis and inhibition of ferroptosis [69]. This finding is in good agreement with the result of an experimental study that showed that dietary cysteine and methionine restriction, together with administration of the GPX4 inhibitor RSL3, enhanced ferroptotic cell death in both murine and human glioma cell lines [71]. Many other genes and proteins involved in the regulation of ferroptosis in gliomas have been discovered, and several combinations of ferroptosis-related genes have been found by gene panel analyses to have a clear correlation with patient prognosis [72,73,74]. Gene set enrichment analysis was performed in 1750 patients with gliomas to analyze the score for enriched genes of different PCD types. The results showed a correlation of this PCD score with an increasing WHO tumor grade of the gliomas and with a shorter survival time of the patients. A second finding was that the PCD score for genes associated with ferroptosis was higher than the score for all other forms of PCD, suggesting a significant role of ferroptosis in glioma malignancy [75]. Furthermore, several studies have investigated the relationship between the expression of individual ferroptosis-associated genes and the prognosis of glioma patients. In addition to confirming the association between poor prognosis of glioma patients and downregulation of ACSL4, overexpression of heat shock protein beta 1 (HSPB1) is also significantly correlated with shorter overall survival, and it is postulated that therapy with the HSPB1 antagonist, ipatasertib, is a promising ferroptosis-inducing therapeutic strategy [73,74]. Non-coding RNA molecules (ncRNAs) have also been shown to influence the induction of ferroptosis, with some ncRNAs being downregulated and others upregulated in gliomas. For all three major types of ncRNAs, which are long non-coding RNAs (lncRNAs), microRNAs (miRNAs), and circular RNAs (circRNAs), several ncRNAs have been described to exhibit either promoter or even suppressor activity in the induction of ferroptosis [76,77,78]. An important example is miRNA-147a, which inhibits SLC40A1-mediated iron export and thus favors ferroptosis-promoting iron overload. Experimental downregulation of miRNA-147a in gliomas promotes SLC40A1-mediated iron export and inhibits ferroptosis [77]. The circular RNA circRNA-RFN5 is downregulated in gliomas, leading to transcriptional upregulation of the ferroptosis suppressor GCH1 [78].

Some authors have discussed that ferroptosis can have different effects on the biological behavior of tumors, depending on the molecular changes and the cell type affected by the activation or inhibition of ferroptosis [62,64,72,79]. An important finding in this context is that the inhibition of SLC7A11, mediated by CD8+ T-cells, triggers ferroptosis and thus cell death of the tumor cells. Conversely, it has been confirmed that ferroptosis leads to the release of damage-associated molecular patterns (DAMPs) such as the oncogene KRASG12D, which are taken up by macrophages in the tumor environment (TEM). This uptake of KRASG12D, in turn, leads to M2 polarization of macrophages and immunosuppression of TEM, which promotes tumor growth [64,79]. T-regulatory cells (Tregs) and myeloid-derived suppressor cells (MDSCs) are two other cellular components of TEM that promote tumor growth by inhibiting the antitumor immunogenic activity of CD8+ T-cells. Tregs and even MDSCs have been shown to be highly resistant to ferroptosis, which is attributed to the activation of the ferroptosis inhibitor GPX4 in Tregs and to the inhibition of TP53 heme oxygenase 1 (HMOX1) in MDSCs [79,80]. This proves that both the ferroptosis resistance of cellular TEM components with immunosuppressive activity and the ferroptosis of cells with immunogenic and tumor-suppressive activity are crucial aspects for understanding the opposing effects of ferroptosis in tumor biology. Evidence for these aspects is provided by a study using a glioma mouse model with the administration of an inhibitor of the checkpoint protein PD-L1 (programmed cell-death-ligand 1) and the administration of the ferroptosis inhibitor ferrostatin 1, either alone or in combination. As a result, only the combined administration of anti-PD-L1 immunotherapy together with the inhibition of ferroptosis by ferrostatin 1 led to a reduced tumor size and a prolonged survival time. As confirmed by immunohistochemistry, the number of CD8+ effector T cells and the immunogenic M1 phenotype of macrophages in tumor tissue increased [75]. In contrast, the tumor suppressive effects of ferroptosis were confirmed in another mouse glioma model using a therapeutic strategy that showed good penetration of the blood–brain barrier by a combination of Fe^3^O^4^ nanoparticles releasing Fe^2+^ and exosomal administration of small interfering RNA molecules suppressing the ferroptosis inhibitor, GPX4. As a result, a clear promotion of ferroptosis together with a significant inhibition of tumor growth was observed [81]. Future efforts to target components of the molecular ferroptosis axis in gliomas must keep this dichotomous relationship between tumor biology and ferroptosis in mind, along with the additional factor of the interrelationship between ferroptosis and other forms of programmed cell death (PCD). In addition to the common molecular pathways in autophagy and ferroptosis as described in the previous chapter, apoptosis and ferroptosis share the common mechanism of being activated by an excess of reactive oxygen species (ROS), and by a positive or even negative regulation due to TP53 activity [69]. The increased lipid peroxidation occurring in ferroptosis is also an activating factor for pyroptosis, while cysteine is an important cofactor for necroptosis but, at the same time, an inhibitory factor for ferroptosis [16,69].

## 4. Pyroptosis

**Figure 3:**
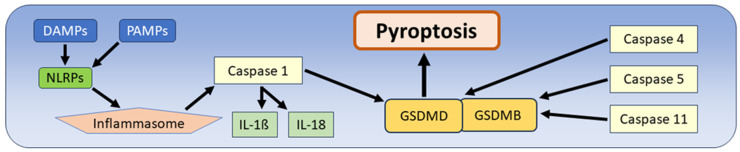
The central signaling pathways of pyroptosis: The innate immune complex (inflammasome) is activated by danger-associated molecular patterns (DAMPs) and/or pathogen-associated molecular patterns (PAMPs) mediated by nucleotide-binding oligomerization domain proteins (NLRPs); activation of the inflammasome leads to activation of caspase 1, expression of the inflammatory cytokines Il-1ß and Il-18 and subsequent activation of gasdermin D (GSDMD), followed by cytokine activation and pyroptosis; an alternative pathway for the initiation of pyroptosis is direct activation of gasdermin D (GSDMD) and/or gasdermin B (GSDMB) by caspases 4, 5, and 11 [14,15,83,84,85].

The first description of pyroptosis in 2001 by Cookson and colleagues provided evidence for a new form of cell death due to Salmonella infection [54]. Intestinal Salmonella infection leads to cell death in multiple host cells, and it was demonstrated that the pathogenicity of the Salmonella-specific islet-1 type III secretion system (T3SS) enabled Salmonella replication in macrophages. Subsequently, cell death of these macrophages occurred due to the activation of caspase-1, followed by the expression of the inflammatory cytokines Il-1ß and Il-18, as well as the porification and dissolution of the cell membranes [13,82]. In further studies, the inflammasome was recognized as a central component of this form of cell death. This is a multiprotein complex containing pattern recognition receptors that can recognize various damage-associated molecular patterns (DAMPs) and even pathogen-associated molecular patterns (PAMPs) present on the surface of pathogenic microorganisms. This recognition triggers an activation cascade of the Toll-like receptor 4 (TLR4) and the nuclear factor kappa-light-chain-enhancer of activated B-cell (NF-kB) complex, which leads to the activation of caspase-1 [83]. The next step in the molecular cascade of pyroptosis is the activation of the gasdermins, which are the enforcers of this type of cell death. Their activation involves cleavage by caspases, which leads to dissociation of the active N-terminal domain [83,84]. In addition to initiating pyroptosis, these highly conserved gasdermin proteins play diverse roles in inflammatory diseases, cancer, and other diseases, and are expressed in various cell types such as epithelia and immune cells. However, it has been emphasized that the exact biological and pathological functions of gasdermins are still largely unknown [14]. In humans, there are six paralogous gasdermins, namely gasdermins A-E (GSDMA-GSDME) and Pejvakin (PJVK). At this point, two different activation modes of pyroptosis must be distinguished. The “classical” pathway of pyroptosis is characterized by the recognition of PAMPs or DAMPs by the inflammasome, the activation of the inflammasome mediated by nucleotide-binding oligomerization domain proteins (NLRPs), the subsequent activation of caspase-1 and the further activation of gasdermin D (GSDMD), which leads to the activation of cytokines, porification of cell membranes and, finally, cell death. The “non-classical” pathway is almost identical, with the exception of the activation of intracellular caspases 4, 5, and 11, leading to activation of gasdermin B (GSDMB) and/or gasdermin D (GSDMD). The subsequent final steps involving cytokine activation and cell death are identical to the classical pathway of pyroptosis (Figure 3) [15,83,84,85]. Because the first characterization of pyroptosis, including its naming, was due to its discovery in Salmonella-infected cells, it has become clear that pyroptosis occurs in various infectious diseases such as inflammation of the respiratory tract or sepsis [86,87]. Pyroptosis also plays an important pathogenic role in other diseases, such as diabetic cardiomyopathy, in which pyroptosis occurs in the hyperglycemic stage [87]. Even neurodegenerative diseases such as Parkinson’s disease have been studied and have shown that inflammasomes and pyroptosis play an important role [14,88,89].

Studies on various types of cancer have confirmed that pyroptosis can play a tumor-promoting or tumor-suppressing role. For most of the 33 cancer types studied by transcriptome analysis of over 10,000 cancer patients, a significant association was found between the level of expression of pyroptosis-related genes (PRGs) and poor patient prognosis. This correlation was confirmed for glioblastomas, low-grade gliomas, clear cell renal carcinomas, and other tumors, such as thyroid carcinomas or prostate carcinomas. In other tumor types, such as bladder carcinoma, pancreatic carcinoma, and cutaneous melanoma, a high expression index for PRGs correlated with a better prognosis and longer overall survival [90]. In transcriptome analyses of gliomas, the expression of several PRGs correlates with the degree of malignancy and inversely with the prognosis of patients. These PRGs, which are upregulated in gliomas, include caspase-3 and caspase-9, n-terminally truncated gasdermin D (GSDMD), and the inflammasome proteins NLRP2 (“NLR family pyrin domain containing 2”) and NLRC4 (“NLR family CARD domain containing 4”). Recent studies have emphasized the lack of in-depth knowledge of inflammasome proteins and their precise molecular interactions. NLRP2 appears to play a role in the regulation of immune responses and in the direct activation of caspase-1, while NLRC4 is associated with the formation of the entire inflammasome [91,92,93]. The increased expression of these caspases and inflammasome proteins confirms the upregulation of pyroptosis and associated genes in gliomas with increasing tumor grade. This is also supported by other studies demonstrating a significant correlation between poor outcome of glioma patients and the expression of caspases 4, 5, 6, and 8, confirming the prognostic importance also of those caspases involved in the “non-classical” pyroptosis pathway (caspases 4 and 5) [94,95,96,97]. Other PRGs that are significantly upregulated in gliomas are tumor protein 63 (TP63), which is known to be associated with cell death and in particular pyroptosis in various tumor types, and neutrophil elastase (ELANE), which is secreted by neutrophils during inflammation and is involved in the progression of various cancers. Another upregulated PRG is nucleotide-binding oligomerization domain-containing protein 1 (NOD1), which is known to promote immunosuppression and tumor progression [91,93]. The influence of a micro-RNA molecule associated with pyroptosis on the biology of gliomas has also been investigated. Micro-RNA 214 (miRNA-214) is significantly downregulated in two glioma cell lines in addition to an upregulation of caspase-1. Experimental upregulation in miRNA-214 inhibited the pyroptosis, cell proliferation, and migration of glioma cells and is therefore considered a target for glioma therapy [98]. Another therapeutic trial is the experimental use of kaempferol, a plant flavonoid with an anti-inflammatory effect. Kaempferol suppressed the proliferation of glioma cells and inhibited tumor growth. At the molecular level, kaempferol led to an increase in ROS, a reduced mitochondrial membrane potential and the induction of autophagy and pyroptosis. Reduced levels of cleaved gasdermin E (GSDME) were observed as a possible explanation for this phenomenon, suggesting that kaempferol induces pyroptosis by regulating autophagy in glioma cells [99]. The small molecule multi-CDK inhibitor AT7519 significantly inhibited the proliferation of glioma cells in vitro. AT7519 inhibited the phosphorylation of CDK1/2, leading to cell cycle arrest, as well as induction of apoptosis and pyroptosis by activation of caspase-3 and cleavage of gasdermin E (GSDME). This induction of cell death via multiple pathways and inhibition of glioma growth adds AT7519 to the list of potential molecular compounds for glioma therapy, and a possible combination with radiotherapy and TMZ chemotherapy remains to be tested, according to the authors [100].

## 5. Necroptosis

**Figure 4:**
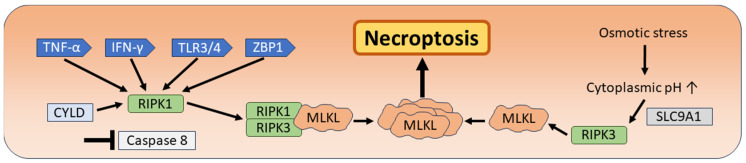
The central signaling pathways of necroptosis: Receptor-interacting protein kinase 1 (RIPK1) can by activated by tumor necrosis factor alpha (TNF-α), or by interferon γ (IFN-γ), toll-like receptors 3 and 4 (TLR3/4), or Z-DNA-binding protein 1 (ZBP1); an inactivated caspase 8 prevents the occurrence of apoptosis; deubiquitination of RIPK1 by the cylindromatosis tumor suppressor protein (CYLD) leads to activation of receptor-interacting protein kinase 3 (RIPK3) and mixed lineage kinase domain-like (MLKL) including its oligomerization, which triggers necroptosis; an alternative activation of RIPK3 occurs through osmotic stress and the consecutive increase in the cytoplasmic pH-value, this form of activation is mediated by the solute carrier family 9 member A1 (SLC9A1) [17,18,19,105,106].

In 2005, Degterev and colleagues observed a form of cell death independent of apoptotic signaling in ischemic brain injury in mice. Morphologically, this form of cell death also differs from apoptosis and resembles necrosis, including swelling of the organelles and rupture of the plasma membrane. Therefore, the term “necroptosis” was proposed [101,102]. Tumor necrosis factor alpha (TNF-α) contributes to the initiation of necroptosis by stimulating tumor necrosis factor receptor 1 (TNFR1), and the small molecule necrostatin-1 has been found to be a potent inhibitor of necroptosis. Necrostatin-1 inhibits the activity of receptor-interacting protein kinase 1 (RIPK1), highlighting the activation of the serine/threonine kinase RIPK1 as an important key molecular step within the activation cascade of necroptosis [101,102,103,104]. Later studies have shown that, in the necroptosis cascade, the initial binding of TNF-α to TNFR1 is followed by the activation of the nuclear factor kappa-light-chain-enhancer of activated B cells (NF-kB) and the ubiquitination of RIPK1, which is regulated by the cellular apoptosis inhibitor protein (cIAP). RIPK1 represents an indispensable step for the further activation of NF-kB and the MAPK pathway involved in cell survival [16]. However, cell death is initiated by the subsequent de-ubiquitination of RIPK1 by the cylindromatosis tumor suppressor protein (CYLD). If caspase-8 is activated during this process, apoptosis occurs. If caspase-8 is inhibited or absent, a “necrosome” is formed, which consists of the receptor-interacting protein kinases 1 and 3 (RIPK1 and RIPK3) and the mixed lineage kinase domain-like (MLKL). RIPK1 binds to RIPK3, leading to the phosphorylation of RIPK3 and the phosphorylation and activation of MLKL, which is then oligomerized and translocated to the cell membrane. This event represents the final step of necroptosis, which leads to an increased permeability of the plasma membrane, causing membrane rupture and the release of DAMPs (danger associated molecular patterns) [16,17,105]. In addition to the first step of TNF-α binding to TNFR1, necroptosis can be triggered by a variety of situations such as ischemia and re-perfusion, calcium overload and heat stress. Osmotic stress directly stimulates RIPK3 kinase activity through an increase in cytoplasmic pH mediated by the Na^+^/H^+^ exchanger solute carrier family 9 member 1 (SLC9A1) [17,18]. Other molecular pathways that can lead to the stimulation of RIPK3 and subsequent necroptosis are the activation of interferon γ-receptors (IFN-γ), toll-like-receptors 3 and 4 (TLR3/4), as well as viral infections leading to activation of the Z-DNA binding protein 1 (ZBP1) (Figure 4) [19,105,106].

A major difference between necroptosis and apoptosis is the close relationship between inflammation and necroptosis, whereas apoptosis induces a lower inflammatory response due to the integrity of the plasma membrane. The close relationship between necroptosis and inflammation is thought to be partly due to the different functions of RIPK1 and RIPK3, which are also involved in inflammatory processes and neuroinflammatory diseases [17,105]. Ongoing research into the functions of the molecular components of necroptosis, and the different ways in which it is triggered, has explained the broad spectrum of diseases in which necroptosis is thought to play a possible role. In the first description of necroptosis, the contribution of necroptosis to ischemic brain damage was confirmed. The mediators of necroptosis also appear to play a functional role in stroke-induced inflammation, including a shift towards the M1 phenotype of macrophages through the influence of RIPK3 and MLKL [101,107]. The role of necroptosis in other diseases such as myocardial infarction, chronic obstructive pulmonary disease, acute pancreatitis, and autoimmune diseases is also currently being investigated [105,108,109].

Studies in different cancer types have shown that necroptosis can have a tumor-promoting or tumor-inhibiting effect depending on the activation mode and tumor type. It has also been found that activation by TNF-α and RIPK1 is not always required for necroptosis of tumor cells in different cancer models. An important example is the finding that glucose deprivation instead of RIPK1 led to activation of MLKL and necroptosis by the release of mitochondrial DNA into the cytoplasm of tumor cells in an experimental model of breast cancer. This type of induction of necroptosis in the necrotic breast tumors was significantly associated with the occurrence of lung metastases [110]. In an experimental melanoma model, necroptosis of endothelial cells from tumor vessels contributed to the extravasation of tumor cells and subsequent metastases [111]. The release of DAMPs by tumor cells undergoing necroptosis has also been shown to contribute to the promotion of angiogenesis, cell proliferation, and metastasis in various tumor types [17]. In contrast, the induction of necroptosis of the tumor cells has shown potent antitumor effects in other experimental tumor models of other cancers and is considered a potential therapeutic strategy, especially for tumors with resistance to apoptosis [17,19]. Nevertheless, the dual role of necroptosis in cancer is frequently addressed, which is also due to its ability to suppress or induce the anti-tumor immune response in the tumor environment (TEM). Research in this field is still ongoing, and the general finding seems to be that chronic necroptosis promotes tumor development due to its suppressive effect on anti-tumor immunity in certain cancers and that the acute induction of necroptosis inhibits tumor growth, including triggering immunogenic responses [16,17,19,106]. Studies on necroptosis and the expression pattern of necroptosis-related genes (NRGs) in gliomas have shown that the expression of NRGs is significantly increased in gliomas compared to control cases and that the activation of necroptosis pathways, reflected by the increased expression of NRGs, correlates with poor prognosis [112,113]. As expected, increased expression in gliomas has also been observed for TNF-α, TNFR1, RIPK1, and RIPK3 [112]. The expression of NRGs also correlates positively with the infiltration of immune cells in TEM and with the expression of checkpoint proteins such as programmed cell death (PD) and programmed cell death ligand 1 (PD-L1) [113,114]. In addition, it has been confirmed that many NRGs are expressed in immune cells and that this expression correlates with poor overall survival. Those tumors with high-risk score and high NRG expression showed strong infiltration of TEM with M2 macrophages, T-regulatory cells (Tregs), and mast cells, which are known to contribute to an immunosuppressive and tumor-promoting phenotype of TEM [114]. As confirmed by gene expression analyses, studies on individual components of the molecular cascade of necroptosis revealed divergent functions, such as a currently unknown functional spectrum for MLKL, which is not only involved in necroptosis, but also in receptor turnover, cytokine secretion, endosomal trafficking, and the formation of extracellular vesicles. In gliomas, a higher expression of MLKL is significantly correlated with poorer prognosis, confirming the view that MLKL and necroptosis play a tumor-promoting role in gliomas as opposed to other tumor types [106,115]. In contrast, the induction of necroptosis in glioma tumor cells under experimental conditions confirmed the antitumor potential of necroptosis. The plant-derived cytokine N6-isopentenyladenosine (iPA) has been used in cancer models due to its antitumor effect, which is based on the disruption of EGFR signaling. In glioma cells, iPA induced necroptosis, whereas no induction of necroptosis was observed in non-neoplastic cell lines. In the glioma tumor cells, iPA led to the activation of RIPK1, RIPK3, and MLDL, but there was no activation of caspases. Treatment of the cells with the necroptosis inhibitor necrostatin-1 inhibited necroptosis, while a general caspase inhibitor had no effect. These results indicate that iPA induces caspase-independent necroptosis of glioma tumor cells [116]. In neurosphere cultures of glioma tumor cells and tumor stem cells, the combined application of the zinc finger transcription factor krüppel-like factor 9 (KLF9) and the histone deacetylase inhibitor panabinostat (LBH589) led to cell death of glioma stem cells by apoptosis and necroptosis. KLF9 is known for its ability to inhibit the self-renewal of glioma stem cells and the growth of tumor xenografts in vivo. Together, KLF9 and LBH589 led to the increased expression of pro- and anti-apoptotic proteins and synergistic cell death, confirming the induction of a caspase-dependent and a caspase-independent pathway thus leading to apoptosis and necroptosis. However, the additional administration of apoptosis inhibitors had only a minor protective effect against cell death, while the administration of necroptosis inhibitors significantly blocked cell death, confirming a significant role of necroptosis in the death of glioma stem cells [117]. In gliomas with IDH mutation, the oncometabolite α-hydroxyglutarate (αHG) is known to promote DNA methylation mediated by methyltransferase 1 (DNMT1). αHG also contributes to promoter methylation of the gene for RIPK3, leading to reduced RIPK3 expression and inhibition of necroptosis of glioma cells. The authors conclude that impaired necroptosis due to αHG-mediated promoter hypermethylation of RIPK3 is an important mechanism in the tumor development of IDH-mutated gliomas [118]. Together with the expression analyses of NRGs, these experimental and in vitro results confirm the dual role of necroptosis for gliomas, depending on the mode of its activation and the target of necroptosis. Further studies on necroptosis in gliomas should include its role in tumor cells and TEM, as well as its regulation by the differential expression of NRGs [106,112,113,115,116,117].

## 6. Cuproptosis

**Figure 5:**
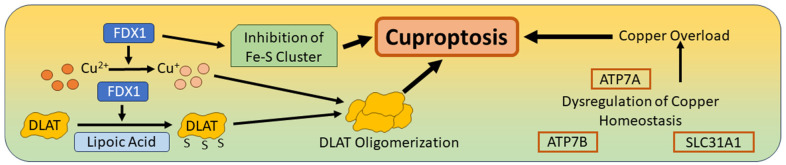
The central signaling pathways of cuproptosis: Copper overload due to dysregulation of copper homeostasis, including the copper transport ATPases, ATP7A and ATP7B, and solute carrier family 31 member 1 (SLC31A1), leads to increased levels of reactive oxygen species (ROS) and cuproptosis; the copper reductase ferredoxin 1 (FDX1) promotes cuproptosis by reducing Cu^2+^ to the more toxic Cu^+^, which leads to the inhibition of iron–sulfur clusters (Fe–S clusters); FDX1 also promotes lipoylation of dihydrolipoamide S-acetyltransferase (DLAT) by lipoic acid, which leads to oligomerization of DLAT and subsequently triggers cuproptosis [20,21,119].

Copper (Cu) is an important cofactor involved in many processes, such as cellular signaling, catecholamine homeostasis, synaptic transmission, myelin formation and the biosynthesis of Cu–zinc superoxide dismutase 1 (SOD1), which is a major component of the cellular antioxidant system. Intestinal copper absorption depends on the presence of Cu transporter 1, and in the bloodstream, Cu is bound to the plasma proteins ceruloplasmin (CP), albumin, and transcopalbumin, which ensure the transport of Cu to peripheral organs and tissues. The Cu-transporting ATPases ATP7A and ATP7B are indispensable for Cu uptake and Cu release. ATP7A ensures the transport of Cu to the liver, which is the most important organ for the excretion and distribution of Cu. In addition, ATP7A facilitates the passage of Cu across the blood–brain barrier (BBB) into the brain [20,119]. Copper-binding small molecules called “copper ionospheres” transport Cu into the cell, and in the cytosol, the copper chaperone of cytochrome c oxidase (COX17) transfers Cu to the mitochondrial membrane, where it is a component of the initiation of respiratory chain enzymes [20,21,119]. In 2022, Tsvetkov and colleagues discovered a new type of cell death triggered by copper overload in a cell line sensitive to the copper ionosphere, elesclomol. This Cu overload induced ROS-dependent cell death without activation of the caspase cascade and without sensitivity to inhibitors of all other known cell death mechanisms. However, cell death due to Cu overload could be blocked by knockout of the gene for the copper reductase ferredoxin 1 (FDX1) and by the knockout of six genes involved in the lipoylation of enzymes of the pyruvate dehydrogenase complex [21]. Lipoic acid is a sulfur-containing fatty acid with antioxidant activity, and the binding of lipoic acid to components of the pyruvate dehydrogenase complex such as dihydrolipoamide S-acetyltransferase (DLAT) is referred to as “lipoylation”. In the presence of excess copper, lipoic acid binds directly to lipoylated DLAT and leads to its oligomerization. In addition, FDX1 reduces Cu^2+^ to the more toxic Cu^+^, leading to the inhibition of Fe–S cluster protein synthesis and induction of cell death, which is termed “cuproptosis” [20,21]. Copper homeostasis mainly depends on the above-mentioned ATPases ATP7A/B and on a third copper transport protein, Solute Carrier Family 31 Member 1 (SLC31A1). Dysregulation of this homeostasis system can lead to copper overload and cuproptosis, but the oligomerization of DLAT and inhibition of Fe–S cluster protein synthesis are also key steps in triggering this newly discovered type of cell death (Figure 5). Excess copper plays an important role in cognitive impairment, as demonstrated in an experimental model by blocking cAMP response element-binding protein (CREB), leading to decreased expression of its downstream target brain-derived neurotrophic factor (BDNF) [120]. Even a significant decrease in the release of the transmitters synaptophysin, dopamine, 5-HT and GABA due to Cu overload has been observed experimentally, leading to memory and learning impairments [97,99]. Research on cuproptosis in neurodegenerative diseases is ongoing, and a recent study found that the expression of cuproptosis-related genes (CRGs) is increased in the peripheral blood of patients with amyotrophic lateral sclerosis (AML) [22].

Despite the short period of time since cuproptosis was first described, a lot of information has already been obtained about its role in tumors. Decreased expression of FDX1 has been found in various tumor types such as renal cell carcinoma, and this decreased expression appears to be closely associated with poorer prognosis [23,121,122]. The expression of SLC31A1 is significantly increased in cancerous tissue compared to non-neoplastic tissue and correlates significantly with shorter overall survival in adrenocortical carcinoma and mesothelioma [123]. Importantly, the correlation of FDX1 expression and SLC31A1 expression with prognosis does not apply equally to all tumor types, and further in vivo and in vitro research is needed to separately confirm the prognostic significance of specific cuproptotic events in different types of cancer [23]. Expression analysis of CRGs in different cancer cells revealed a significant correlation among CRG expression levels, the activation of glutamine metabolism, and the p53 signaling pathway [124]. In gliomas, gene expression analysis confirmed a correlation between the expression of CRGs and genes involved in glutaminase activity, which also correlated with a poorer prognosis of glioma patients and with the extent of immune cell infiltration in the tumors. Glutaminase is an amidohydrolase that catalyzes the hydrolytic deamination of glutamine to glutamate, which is subsequently transferred to the tricarboxylic acid cycle (TAC). Gene expression analysis also showed a strong correlation of CRG expression with genes related to glucose metabolism and with the degree of immune cell infiltration of gliomas. The authors hypothesized that glutaminase activity and its close association with immune cells and glucose metabolism may mediate cuproptosis in gliomas in a previously unknown manner [125]. Several gene expression analyses independently confirmed increased expression of the FDX1 gene in gliomas compared to healthy tissue, and this expression correlated significantly with a poorer clinical prognosis [126,127,128]. This prediction of poorer prognosis was also independently confirmed for high-grade gliomas and for low-grade gliomas [122,128], while in renal cell carcinomas, the opposite result was confirmed and FDX1 expression correlated with a better prognosis [122]. FDX1 expression also correlated with the amount of immune cell infiltration in TEM, especially with the number of macrophages and myeloid-derived suppressor cells, which are known to contribute to immunosuppression of TEM and promotion of tumor growth [127,128]. Another significant correlation exists between the expression of FDX1 and the autophagy marker genes ATG5, Beclin1 (ATG6), and ATG12, suggesting an interaction between FDX1-mediated cuproptosis and autophagy. It is hypothesized that autophagy is also a pre-stress process of cuproptosis, and that cells that fail to regulate cellular homeostasis through autophagy may switch to the mechanism of cuproptotic cell death [129]. The expression of epiregulin (EREG) was also closely correlated with the expression of CRGs. EREG is a member of the epidermal growth factor family and a ligand of the epidermal growth factor receptor (EGFR). It has multiple functions and is involved in angiogenesis, inflammation, and stimulation of cell proliferation. In gliomas, EREG expression is closely correlated with the expression of the checkpoint protein PD-L1 and inversely correlated with the expression of FDX1, which is related to cuproptosis. The authors therefore hypothesize that EREG is an oncogenic protein that can influence immunity and cuproptosis by affecting the expression levels of PD-L1 and FDX1 [130]. Two other checkpoint proteins, CD24 and CD47, were also correlated with the expression of CRGs and their expression was significantly increased in gliomas. Both checkpoint proteins are known to correlate with the degree of malignancy of tumors and with the degree of immune cell infiltration in tumor tissue [131]. The same was confirmed for the expression of SLC31A1, one of the main proteins responsible for copper uptake. SLC31A1 gene expression correlates positively with immunosuppressive M2 macrophage infiltration, but negatively with pro-inflammatory M1 macrophage infiltration, which may lead to the formation of an immunosuppressive TME in gliomas. Furthermore, the expression of SLC31A1 was increased in gliomas compared to healthy tissue, and the expression correlated significantly with a worse prognosis. This finding is consistent with the results also confirmed in adrenocortical carcinoma and mesothelioma [123,132]. Although SLC31A1 is involved in copper metabolism and is significantly associated with the prognosis and biological behavior of gliomas, the authors point out that its exact role in cuproptosis is still unclear and requires further investigation [132].

## 7. Other Forms of Programmed Cell Death

### 7.1. Paraptosis

The morphological features of paraptosis are the presence of cytoplasmic vacuoles and the absence of changes characteristic of apoptosis, such as the fragmentation of nuclei, or the fragmentation of chromatin or apoptotic bodies. The vacuoles differ from the morphology of double-membrane-coated autophagosomes in autophagy, thus swelling of the endoplasmic reticulum (ER) and mitochondria may occur. These findings were already mentioned in the first description of paraptosis by Sperandio [24,25,133]. Paraptosis shows no response to caspase inhibitors, but one activating factor is caspase 9, which is independent of apoptotic peptidase activating factor 1 (APAF1) activity. Caspase 9 can induce apoptosis and paraptosis, while apoptosis can be inhibited by blocking apoptotic peptidase-activating factor 1 (APAF1) using the apoptosis inhibitor T-butyloxycarbonyl-Asp(O-methyl)-fluoromethyl ketone (BAF). Another paraptosis-activating factor is human insulin-like growth factor 1 receptor (IGFIR), which triggers non-apoptotic cell death that fulfills the morphological criteria of paraptosis mentioned above, and this effect of IGFIR is not cell type specific [24]. The process of paraptosis is mediated by the mitogen-activated protein kinase pathway (MAPK), which has been confirmed by the experimental inhibition of mitogen-activated protein kinase 2 (MEK2) and Jun-N-terminal kinase 1 (JNK). Triggering paraptosis via the mitogen-activated protein kinase (MAPK) pathway includes the proteins rat sarcoma protein family (Ras), rapidly accelerated fibrosarcoma (Raf), mitogen-activated protein kinases 1 and 2 (MEK1/2), and extracellular signal-regulated kinases 1/2 (ERK1/2) [25].

**Figure 6:**
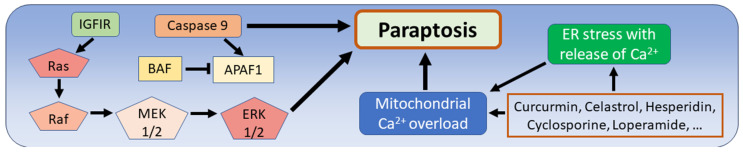
The central signaling pathways of paraptosis: Insulin-like growth factor 1 receptor (IGFIR) is an important activating factor that triggers paraptosis by the mitogen-activated protein kinase (MAPK) pathway, including the proteins rat sarcoma protein family (Ras), rapidly accelerated fibrosarcoma (Raf), mitogen-activated protein kinases 1 and 2 (MEK1/2), and extracellular signal-regulated kinases 1 and 2 (ERK1/2); caspase 9 can induce apoptosis and paraptosis, while apoptosis can be inhibited by blocking apoptotic peptidase-activating factor 1 (APAF1) using the apoptosis inhibitor T-butyloxycarbonyl-Asp(O-methyl)-fluoromethyl ketone (BAF); several other natural and synthetic compounds can induce paraptosis by inducing stress in the endoplasmic reticulum with release of Ca^2+^ and mitochondrial calcium overload [24,26,134,135].

The multifunctional protein ALG-2-interacting protein 1 (AIP1) has been described as a specific inhibitor of paraptosis, which is involved in various functions such as the regulation of the cytoskeleton in different species, although the exact functional spectrum of AIP1 is not yet fully understood [24,25]. In addition to IGFIR, several other factors have been described to promote paraptosis, such as vanilloid receptor subtype 1 (VR1), neurokinin-1 receptor (NK1R), epidermal growth factor (EGF), 1-nitropyrene (1-NP), excess ROS, and increased cellular and mitochondrial Ca2^+^ influx [26]. This Ca2^+^ imbalance is the main mechanism triggered by many compounds recognized as promoters of paraptosis, including natural phenols or other organic substances such as curcumin, celastrol and hesperidin. Many other substances such as cyclosporin A, oligomeric procyanidins, or loperamide have shown a paraptosis-promoting effect in experimental and in vitro studies (Figure 6) [26,134,135]. In contrast to ferroptosis, which has opposite effects on tumor biology as described in the previous chapters, current research considers paraptosis as a general tumor-limiting phenomenon, which has been confirmed for various cancers with the inhibition of paraptosis in experimental settings [26]. Even for gliomas, the studies performed so far have shown non-apoptotic cell death that fulfills the criteria of paraptosis by blocking various proteins involved in energy metabolism. This applies to the inhibition of adenine nucleotide translocase 1 (ANT1), which catalyzes the final step of oxidative phosphorylation, i.e., the exchange of ATP produced in the mitochondria by ATP synthase with ADP produced in the cytosol [136]. Other targets are cyclophilins, a group of proteins that are involved in immunomodulation and energy metabolism and are known to have tumor-promoting properties. Their targeting by the small molecule cyclophilin inhibitor NIM811 led to paraptosis of glioma tumor cells [137]. Direct experimental induction of paraptosis of glioma cells could be achieved by various compounds, such as curcumin, which is known to regulate the cell cycle and activate TP53 [138,139], and the phytotoxin ophiobolin A, which is known to have anti-cancer properties [140,141]. In addition, the natural alkaline aloperine, known for its ability to arrest the cell cycle and suppress tumor growth, can induce the phenomenon of paraptosis, as demonstrated in various cancers [142]. The authors of studies on gliomas and human diseases in general agree that the exact pathways of paraptosis remain to be largely elucidated [26,133,135].

### 7.2. Methuosis

**Figure 7:**
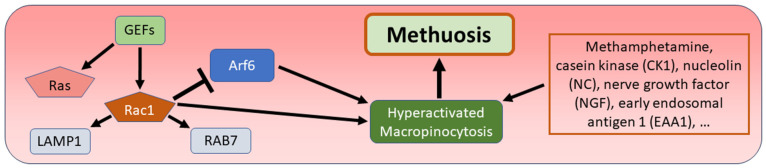
The central signaling pathways of methuosis: The initiation of methuosis begins with the activation of rat sarcoma protein family (Ras) and Rac family small GTPase (Rac1) by guanine nucleotide exchange factors (GEFs), followed by inactivation of ADP-ribosylation factor 6 (Arf6), leading to continuous swelling of macropinosomes and hyperactivation of macropinocytosis; other endosomal markers present in macropinosomes are lysosome-associated membrane protein 1 (LAMP1) and Ras-related protein member 7 (RAB7); a growing number of exogenous and endogenous compounds are described that promote hyperactivation of macropinocytosis and lead to methuotic cell death [143,144,145,146,147,148].

According to the first description by Overmeyer and colleagues, the term “methuosis” is a neologism derived from the Greek “methuo”, which means “to become intoxicated” [143,144]. This neologism refers to hyperactivated macropinocytosis, which is the main characteristic phenomenon of methuosis. In contrast to micropinocytosis, macropinocytosis is a clathrin-independent process that leads to vesicles with a diameter between 0.2 and 5 µm, which are called macropinosomes [145]. The molecular initiation of methuosis begins with the activation and overexpression of the G-protein Ras (“rat sarcoma”) and the downstream G-protein Rac1 (“Rac family small GTPase 1”) by guanine nucleotide exchange factors (GEFs), followed by the inactivation of Arf6 (“ADP ribosylation factor 6”). These proteins are involved in the activation of macropinocytosis and endosomal recycling. Blocking Arf6 hinders the process of macropinosome recycling and the process of their fusion with lysosomes, which leads to continuous swelling of macropinosomes with bursting of the plasma membrane, cell lysis, and cell death [143,144,145,146,147,148]. Other typical features of methuosis are the presence of the late endosomal markers “lysosome-associated membrane protein 1” (LAMP1) and “Ras-related protein member 7” (RAB7) in macropinosomes, the independence of methuosis from molecular components of apoptosis such as caspases, and the lack of involvement of the PI3K signaling pathway and the classical Ras–Raf–MEK axis of the MAPK signaling pathway [27,144]. Macropinocytosis is a non-selective endocytic process for the uptake of extracellular substances and has a dual role in cancer. It can promote tumor growth by increasing extracellular nutrient delivery or by transporting receptors such as platelet-derived growth factor receptor (PDGFR) or epidermal growth factor receptor (EGFR), which activate signaling pathways in tumors. However, in its ultimately activated state, macropinocytosis induces tumor cell methuosis, which is generally considered a tumor-suppressive phenomenon [28,29]. The first described promoter of methuosis was methyl-1H-indole-pyridinyl-propene-1 (MIPP), a molecule that resembles the structure of a group of naturally occurring flavonoid precursors called chalcones. A variant of MIPP called methoxy-methyl-1H-indole-pyridinyl-propene-1 (MOMIPP) was able to trigger methuosis at a much lower concentration [144,149]. Since then, a growing number of other methuosis-inducing compounds have been discovered, including methamphetamine, casein kinase 1 (CK1), nucleolin (NC), nerve growth factor (NGF) and early endosomal antigen 1 (EAA1) (Figure 7) [27,148]. Recent studies on glioma cell methuosis have the common main goal of investigating the molecular action of methuosis-initiating agents. MOMIPP led to early disruption of glucose uptake and glycolytic metabolism as well as activation of the JNK1/2 stress signaling pathway with consecutive phosphorylation of pro-survival members of the BCL2 family. In addition, rapid penetration of MOMIPP through the blood–brain barrier (BBB) with suppression of intracerebral glioblastoma xenografts was demonstrated in a mouse model [150]. An ultrastructural investigation of the effect of MOMIPP showed impaired vesicular traffic between macropinosomes and their surroundings and confirmed the absence of fusions between macropinosomes and lysosomes in glioma cells [151]. For the quinolone derivative vaquinol-1 (Vac), involvement of caspases 3 and 7 in the process of methuosis of glioma cells was demonstrated. However, it was explicitly mentioned that these caspases are not essential for methuotic cell death [152]. Of great clinical importance is the observation that the three methuosis-inducing compounds MOMIPP, vaquinol-1, and honokiol increased the uptake of temozolomide by glioma cells in vitro [153].

### 7.3. Parthanatos

**Figure 8:**
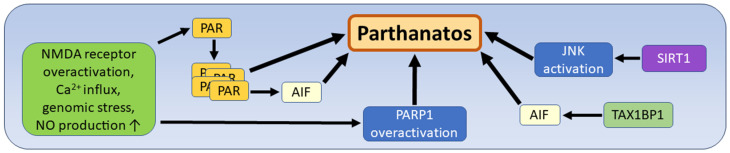
The central signaling pathways of parthanatos: Severe neuronal stress or neuronal injury that leads to NMDA receptor overactivation, increased cellular calcium influx, genomic stress and overproduction of nitrite oxide (NO) promote the overactivation of poly-ADP-ribose polymerase-1 (PARP-1), which is the main trigger of parthanatos; in addition, the nuclear protein poly-ADP-ribose (PAR) forms polymers that lead to increased mitochondrial release of apoptosis-inducing factor (AIF), which is another factor that promotes the induction of parthanatos; several other promoting factors have been described, such as Sirtuin 1 (SIRT1) via activation of the c-Jun N terminal kinase (JNK) pathway and Tax 1-binding protein 1 (TAX1BP1) via upregulation of AIF [30,31,161,162].

Another type of cell death, called “parthanatos”, was first described by Andrabi and coworkers in 2008 and is defined as a cell death that is largely dependent on an excess of poly-ADP-ribose polymerase-1 (PARP-1). The neologism, “Parthanatos”, is a combination of the first three letters of the abbreviation PARP-1 and the Greek word for the personified mythological death, “Thanatos” [154,155]. PARP-1 is a nuclear enzyme involved in the maintenance of cellular homeostasis and DNA repair. It belongs to the superfamily of PARP proteins that catalyze the post-translational ADP-ribosylation of chromatin proteins and varies in its cellular localization. The cellular function of PARP-1 ranges from supporting survival to cell death, and under physiological conditions, mild genomic stress leads to PARP-1 activation and repair of damaged DNA. However, severe neuronal stress or injury triggers the cascade of N-methyl-D-aspartate (NMDA) receptor overactivation, leading to cellular calcium influx, increased activation of neuronal nitrite oxide synthase (nNOS), and overproduction of nitrite oxide (NO). As a downstream effect of increased NO production, the enzyme PARP-1 is overactivated, leading to cell death. Morphological features of this type of cell death, termed parthanatos, are shrunken and condensed nuclei and disintegration of cell membranes, and the main molecular mechanisms are the increased consumption of NAD+ and the depletion of ADP due to the increased activity of PARP-1 [30,142]. Two additional features induced by PARP-1 overactivity are part of the typical cascade leading to this specific type of cell death. First, the nuclear protein poly-ADP-ribose (PAR) is translocated from the nucleus to the cytosol, where it forms polymers and acts as a toxic stimulus for DNA damage. As a direct effect of the polymerization of PAR molecules, there is an increased mitochondrial release of apoptosis-inducing factor (AIF) and its translocation from the cytosol to the nucleus, leading to chromatin condensation. The involvement of parthanatos in various forms of cancer and several diseases of visceral organs and the retina has been confirmed with polymerization of PAR being the central mechanism in the induction of parthanatos [30,31]. In glioblastomas, PARP-1 is expressed in the tumor cells, but not in normal neurons from controls or from tissue outside the tumor [156]. The expression level of PARP-1 is generally higher in glioma cells than in normal cells and is also positively correlated with the malignancy of the tumors and inversely correlated with the prognosis of the patients [157,158]. Parthanatos in gliomas has been studied experimentally and in vitro using the natural substance deoxy-podophyllotoxin (DPT), which is known to induce this type of cell death. As an important additional insight into the molecular cascade of parthanatos, DPT-induced upregulation of sirtuin-1 (SIRT1) was found to lead to upregulation of the JNK pathway, which, in turn, contributes to increased oxidative stress and reactive oxygen species (ROS) formation [159,160,161]. Another important detail of parthanatos is Tax 1-binding protein 1 (TAX1BP1), which is upregulated by DPT, leading to the nuclear translocation of AIF and parthanatos of glioma cells (Figure 8). This effect is thought to be due to downstream activation of TAX1BP1 following the upregulation of PARP-1, leading to the upregulation of mitochondrial respiratory chain complex 1 by TAX1BP1 [162]. The exact role of parthanatos in gliomas and even the spectrum of its pathways and activation modes are still not clear. Especially the exact role of AIF in parthanatos and the divergent activity of PARP-1, ranging from its role in cellular survival to cell death, are the subject of further research [30].

### 7.4. Anoikis

**Figure 9:**
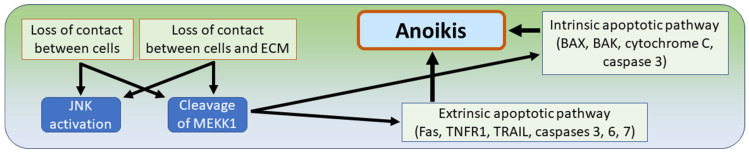
The central signaling pathways of anoikis: Loss of contact between neighboring cells or between cells and extracellular matrix (ECM) leads to activation of the c-Jun N terminal kinase (JNK) pathway and activation and cleavage of the intracellular kinase mitogen-activated protein kinase kinase-1 (MEKK-1); these events promote the typical pathways of apoptosis leading to cell death; in general, anoikis is characterized by the two signaling pathways of apoptosis (intrinsic and extrinsic) triggered by loss of cell-cell contact or cell-ECM contact [32,33,163].

Frisch and Francis introduced the term “anoikis” in the year 1994 for apoptotic cell death triggered by the disruption of interactions between normal epithelial cells and the extracellular matrix. As mentioned in the initial description, the Greek term “anoikis” stands for the state of being without a home [163]. Anoikis has the same characteristics as apoptosis, including the typical morphology with nuclear fragmentation, as well as the same molecular pathways. These include the intrinsic pathway, which is based on the oligomerization of the pro-apoptotic proteins BAX (“BCL2-associated X protein”) and BAK (“BCL2-homologous antagonist”) at the outer mitochondrial membrane, leading to the release of cytochrome C and the subsequent activation of the effector caspase 3. The second (extrinsic) pathway is initiated by members of the tumor necrosis factor receptor (TNFR) superfamily such as the Fas receptor, tumor necrosis factor receptor 1 (TNFR1), and tumor necrosis factor-related apoptosis-inducing ligand (TRAIL), leading to the activation of caspase 8 and the subsequent cleavage and activation of effector caspases 3, 6, and 7 [32,33,163]. Since the molecular cascade of anoikis has clearly identical features compared to apoptosis, the remaining main difference is the initiation of anoikis after disruption of the cellular contact to neighboring cells and/or the extracellular matrix. Activation of the Jun N-terminal kinase (JNK) pathway is a key step leading to the promotion of anoikis [32]. The activation of anoikis is further supported by cleavage of the intracellular kinase mitogen-activated protein kinase kinase-1 (MEKK1), which occurs in cells that lose contact with the matrix. Subsequently, the overactivation of the cleavage form of MEKK1 leads to anoikis (Figure 9) [32]. A second important topic is the search for molecular components of signaling pathways that lead to tumor resistance to anoikis. Various types of cancer are currently under investigation, and different proteins and non-coding RNA molecules have been shown to be involved in the cascade of anoikis resistance in various tumors such as prostate cancer and renal cell carcinoma [164,165]. In gliomas, an important factor of anoikis resistance is elongation factor-2 kinase (eEF2), which contributes to the malignant phenotype of glioblastomas by promoting glioma cell migration and invasion. Experimental silencing of eEF2 kinase resulted in the inhibition of cell migration and reduction in cell attachment, along with induction of anoikis [166]. Expression of the transcription factor motor neuron and pancreatic homeobox 1 (MNX1) correlates with the degree of malignancy of glioma cells. Overexpression of MNX1 and tyrosine kinase receptor B (TrkB) reduces tumor cell adhesion, leading to suppression of anoikis. Therefore, MNX1 is considered a potential target for glioma therapy to achieve anoikis induction [167]. P21-activated kinase 4 (PAK4) is also a promoter of glioma growth. The involvement of PAK4 in anoikis resistance was confirmed by its experimental silencing, which led to increased anoikis and inhibition of glioma cell invasion and migration through downregulation of metalloprotease 2 (MMP2), alpha-v beta-integrin (Avß3) and epidermal growth factor receptor (EGFR) [168]. Another protein called melanoma differentiation associated gene-9/syntenin (MDA9) has been shown to maintain protective autophagy in glioma stem cells through EGFR signaling and phosphorylation of BCL2. Experimental downregulation of MDA9 in anoikis-resistant glioma cells resulted in sustained levels of autophagy and inhibition of BCL-2 phosphorylation, leading to anoikis of tumor stem cells. The authors address that MDA9-targeted therapy could potentially be used clinically as part of a combinatorial approach with chemotherapy or radiotherapy, both of which often utilize protective autophagy in their resistance mechanisms [169]. Other authors have supported the hypothesis that anoikis resistance is involved in the development of resistance to radiotherapy and even chemotherapy; however, they have also argued that anoikis resistance in gliomas has not yet been extensively studied [170]. A groundbreaking observation is the overexpression of RGD-binding integrins in malignant gliomas that recognize the arginine–aspartic acid–glycine (RGD) sequence in the extracellular matrix. It is known that these integrins play a supporting role in the proliferation of glioma stem cells. Treatment of stem cells with the small molecule RGD antagonist 1α-RGD resulted in anoikis, supporting the view that therapeutic application of 1α-RGD may represent another potential pharmacological approach to induce anoikis of glioma stem cells [171,172].

## 8. Discussion

For a long time, the term, “apoptosis”, was considered synonymous with the term “programmed cell death”. In contrast, the term, “cellular necrosis”, stands for the non-programmed and pathological form of cell death due to external or internal stimuli. Since the first description of apoptosis in 1972 by Kerr and colleagues [173], the two signaling pathways and the significance of apoptosis in various diseases have been intensively researched. The “extrinsic” signaling pathway is mediated by receptors of the tumor necrosis factor family (TNF) such as TNFR1 or TRAIL, as well as by effector caspases 3, 6, and 7. The “intrinsic” (mitochondrial) signaling pathway is mainly characterized by the proapoptotic proteins, BAX and BAK, the mitochondrial release of cytochrome C, and by effector caspase 3 [174,175]. In gliomas, apoptosis has a diverse significance, and the most important aspects include the prevention of apoptosis in endothelia in the context of tumor-associated neo-angiogenesis [176], as well as the induction of apoptosis of immunocompetent cells with tumor-inhibiting capacity such as natural killer cells and T lymphocytes in the tumor environment [177]. Currently, research into cellular molecular components that contribute to the prevention of apoptosis of tumor cells and the associated resistance to temozolomide therapy is particularly noteworthy. These include the ubiquitin-conjugating enzyme E2C (UBE2C), the so-called “outer dense fibers of sperm tail 3B” (ODF3B), which is expressed in many tumor types, as well as the PDZ domain-containing protein 1 (PDZK1) [178,179,180]. In contrast to earlier decades, the term “programmed cell death” now represents a generic term for the entirety of all molecularly defined forms of cell death. In addition to apoptosis, which has long been the subject of intensive research in gliomas, it was therefore necessary to provide a detailed overview of the current state of knowledge on all forms of non-apoptotic PCD in individual tumor types such as gliomas. Compared to apoptosis, it still seems justified to describe these other forms of PCD as “new”. All the described forms definitely represent different pathways of cell death, as they all differ from each other by a characteristic molecular cascade with the activation of specific proteins and molecular components. As described in the individual chapters, many forms of PCD show frequently occurring cytological changes that distinguish them from apoptosis, but it is important to emphasize that morphological changes alone do not allow for a reliable differentiation of the forms of PCD. Therefore, an analysis of the characteristic molecular changes is a prerequisite for the detection of the respective forms of PCD using various methods such as immunohistochemistry and immunofluorescence, polymerase chain reaction and/or microarray systems and, if necessary, quantitative image analysis, flow cytometric quantification, or even gene expression analyses [26,44,71,125].

**Figure 10:**
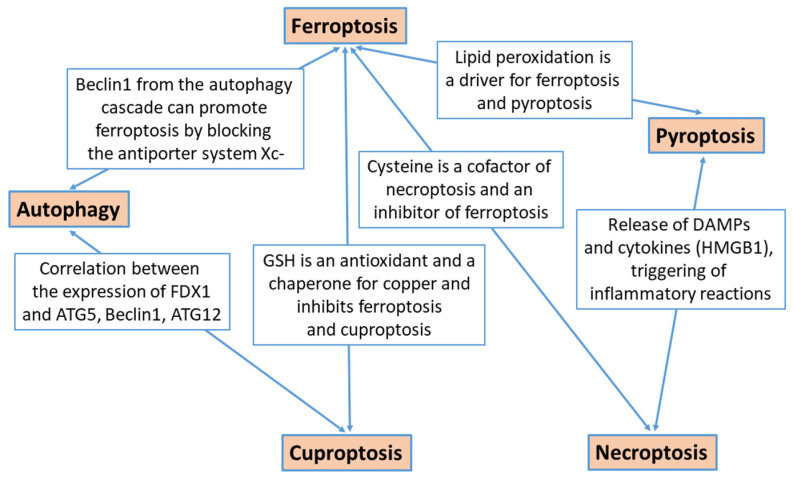
Molecular interactions between different forms of non-apoptotic cell death—important aspects as presented in the main text [16,61,69,129].

**Figure 11:**
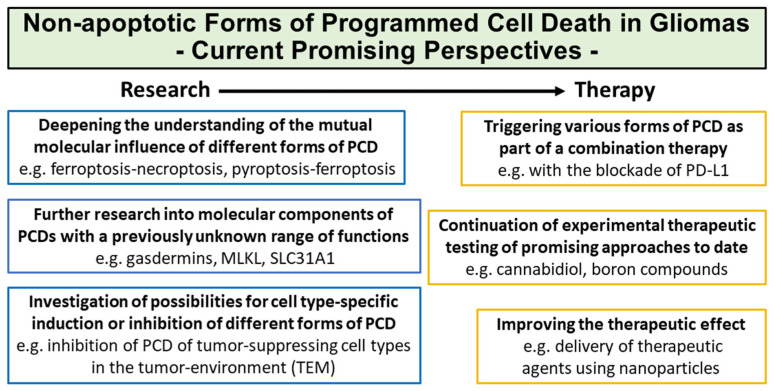
Important current perspectives for the research and therapeutic targeting of non-apoptotic forms of programmed cell death (PCD) in gliomas. In addition to the need for a deeper understanding of the mutual interaction of different PCDs and for molecular components with a previously unknown range of functions, research into the possibilities of cell-type-specific induction of PCDs is also a promising perspective [16,69,79,80,81,106,132]; important therapeutic perspectives include the possibility of inducing PCDs as part of a combination therapy, the use of nanoparticles for the purpose of improved delivery of therapeutic agents, and the continuation of research into promising therapeutic approaches, for example, using cannabidiol or boron compounds [75,181,182,183].

A fundamental finding is the different biological significance of individual forms of non-apoptotic PCD in different tumor types, which is reflected, for example, in the fact that the expression of certain genes associated with certain forms of PCD is significantly associated with a longer survival time in some tumor types and with a poorer prognosis in other tumor types. One example is the expression of pyroptosis-related genes (PRGs), which are associated with a poorer prognosis in gliomas and in carcinomas of the kidney and prostate, but with a better prognosis in carcinomas of the urinary bladder and pancreas [90]. Another example is the increased expression of the gene for the copper reductase ferredoxin 1 (FDX1) in gliomas compared to healthy tissue, which correlates significantly with a poorer prognosis for patients [126,127,128]. The opposite result was found in renal cell carcinomas, where increased expression of FDX1 correlated with a better prognosis, demonstrating a different role of cuproptosis-inducing FDX1 in different tumor types [122]. In gliomas, gene expression analyses revealed a significant correlation between the expression of genes related to individual forms of PCD and a poorer prognosis of the patients for most non-apoptotic forms of PCD, namely for ferroptosis [75], pyroptosis [90], necroptosis [112,113], cuproptosis [122,128], and parthanatos [157,158]. The phenomenon of autophagy represents a special case because the amount of expression of autophagy-related genes (ATGs) also correlates with a poor prognosis of patients with gliomas [55], which is in line with experimental studies confirming the tumor-promoting properties of autophagy [44,45,46]. In contrast, the induction of autophagic cell death, by means of experimental methods for example, has been shown to exhibit a tumor-suppressing effect in gliomas [49,50,51]. The tumor-promoting effect of the enhanced expression of genes related to autophagy and other forms of PCD has been shown to correlate with an immunosuppressive tumor environment. It seems likely that this chronic activation of the forms of PCDs plays a major role in the induction of this tumor-promoting environment due to the support of phenotypic alterations of macrophages or other immunogenic cells such as CD8+ T-cells [54,55,75,96,113,127,128,129]. However, since the spontaneous and, so far, mostly experimental induction of these forms of PCD has led to cell death of the tumor cells and thus has a tumor-suppressive effect in most cases, it seems justified to transfer an assumption already formulated for necroptosis to the other forms of PCD mentioned. This assumption says that chronic induction of these PCD forms promotes tumor development due to their suppressive effect on anti-tumor immunity, and that the acute induction of these PCD forms inhibits tumor growth due to the induction of tumor cell death [16,17,19,106]. Undoubtedly, further detailed research is needed on numerous detailed questions and an important aspect in this context is the need for the further investigation of the molecular interactions between the different non-apoptotic forms of PCD. Important findings have already been made which show that the forms of PCD are significantly interrelated at the molecular level and thus that the isolated consideration of individual PCDs is less meaningful in assessing their role in gliomas than their totality. As mentioned in the previous chapters, there are important molecular interactions between ferroptosis and other PCDs. These include the possibility of promotion of ferroptosis by the autophagic protein Beclin1 [61], the inhibitory effect of glutathione (GSH) on both ferroptosis and cuproptosis [69], and the importance of cysteine, which is both an inhibitory factor for ferroptosis and a cofactor for necroptosis [16,69]. Increased lipid peroxidation is a major driver for the induction of ferroptosis and pyroptosis [69]. Another important example of the molecular interaction of PCDs is the correlation of the expression of the cuproptosis-inducing protein ferredoxin 1 (FDX1) and the autophagic proteins ATG5, Beclin1, and ATG12 [129]. The similarity and molecular interaction of pyroptosis and necroptosis can be seen in the expression of damage-associated molecular patterns (DAMPs) and inflammation-promoting cytokines by tumor cells such as high mobility group box 1 (HMGB1), which is associated with an increase in antigen processing of immune cells [16] (Figure 10). Another important aspect is to gain more insight into the molecular components of non-apoptotic PCD forms with a previously unknown functional spectrum. As mentioned in the corresponding chapters on the individual forms of PCD, this applies to the family of gasdermins, whose functions go far beyond their contribution to pyroptosis [14]. It also applies to the mixed lineage kinase domain-like protein (MLKL), which is a multifunctional protein and not just an enforcer of necroptosis [106], and the copper transport protein solute carrier family 31 member 1 (SLC31A1), which shows increased expression in gliomas, but whose exact role in cuproptosis still needs to be investigated [132]. An additional significant aspect in glioma research is the investigation of exosomal delivery of molecular components influencing the forms of cell death and the formation of an immunosuppressive TEM. An important observation in this context is the proof of the initiation of autophagy and M2-polarization of macrophages by glioma-derived exosomes thus leading to glioma progression. Interleukin 6 (IL-6) and micro-RNA miR-155-3p were highly expressed in these exosomes and are considered the main components that trigger autophagy and an immunosuppressive phenotype of macrophages [52]. Many more studies have revealed that non-coding RNA molecules like micro-RNAs can influence different forms of PCD in gliomas, and further studies on their activity and delivery through exosomes are worthwhile [76,77,78,81,98]. The necessity for these studies also arises from the fact that many cells with immunosuppressive and thus tumor-promoting properties are resistant to forms of PCD, such as Tregs and MDSCs, which are highly resistant to ferroptosis. In addition to further research into the associated signaling pathways and potential therapeutic approaches, it would also appear to make sense to consider the possibilities of cell-type-specific initiation of forms of cell death in the future [79,80] (Figure 11). Plant components are also currently playing an increasingly important role in efforts to induce non-apoptotic forms of PCD in gliomas with the aim of tumor suppression. Two important examples are the plant-derived flavonoid, kaempferol, which leads to induction of autophagy and pyroptosis of glioma cells [99], and the plant-derived cytokine N6-isopentenyladenosine (iPA), which leads to activation of RIPK1, RIPK3, and MLDL, with subsequent induction of necroptosis [116]. At present, many new findings on the non-apoptotic forms of PCD should be mentioned, confirming a partly tumor-promoting and partly tumor-suppressive effect for most forms. Important exceptions are paraptosis, methuosis, and anoikis, for which a predominantly tumor-suppressive effect in gliomas has been confirmed so far. Further investigation of this aspect should also be seen as an important perspective for future glioma research [26,142,150,153,169,172].

In addition to the aforementioned forms of cell death, other forms have been described that also play a role in various diseases and tumors but, with the exception of panoptosis, are still largely unexplored in gliomas. These additional forms of cell death include NETosis, oxeiptosis, entosis, and panoptosis. NETosis refers to a phenomenon initiated by neutrophil granulocytes forming net-like structures called neutrophil extracellular traps (NETs), which consist of cellular components such as histones, DNAm and neutrophil cytoplasmic proteins such as cathepsin D and myeloperoxidase. These NETs can be triggered by various intracellular or extracellular pathogens and are capable of killing or capturing these pathogens, including bacteria, viruses, or fungi. In addition, NETs can also be induced by immune complexes, cytokines, chemokines, or other physiological stimuli [34,35,184]. There are two forms of NETosis, namely “vital NETosis”, which does not lead to cell death, and “suicidal NETosis”, which is dependent on an increase in calcium concentration and the subsequent activation of the protein kinase C (PKC) pathway and the NADPH oxidase complex (NOX). This suicidal NETosis leads to the disintegration of cytoplasmic and nuclear components, followed by caspase-independent cell death. NETosis has been observed in various immune diseases such as systemic lupus erythematosus and rheumatoid arthritis, but also in tumor cells and in cells from the tumor environment. It is assumed that the hypoxic environment with increased levels of hypoxia-inducible factor 1α causes NETosis in tumors [34]. In an experimental study, NETosis was investigated in gliomas treated with an oncolytic herpes simplex virus (oHSV). This treatment led to increased overexpression of insulin-like growth factor 2 mRNA-binding protein 3 (IGF2BP3), a carcinoembryonic protein that is overexpressed in many tumors such as malignant gliomas. This overexpression led to the induction of NETosis in tumor cells and the attenuation of the oncolytic capacity of oHSV therapy, suggesting NETosis as a target in gliomas as a strategy to overcome resistance in oncolytic virotherapy [185]. Oxeiptosis is a form of cell death that is triggered by the interaction of reactive oxygen species (ROS) with the most important intracellular ROS sensor called “kelch-like enoyl-CoA hydratase-associated protein 1” (KEAP1). A mediator protein of various forms of cell death is phosphoglycerate mutase 5 (PGAM5), which interacts in oxeiptosis with KEAP1 and apoptosis-inducing mitochondria-associated factor 1 (AIFM1), which is dephosphorylated by PGAM5 at position S116. This dephosphorylated site S116 of AIFM1 is a prerequisite and thus a marker for the consecutive occurrence of the caspase-independent form of cell death, the so-called “oxeiptosis”. Research into the occurrence of oxeiptosis in various diseases has only just begun, but even in the initial description, it was suggested that oxeiptosis could be influenced by therapeutic intervention in diseases associated with oxidative stress [36]. In patients with hepatocellular carcinoma (HCC), screening of oxeiptosis-related genes has provided a statistical prognostic system for HCCs [186]. In patients with glioblastoma, a global screening of all known genes associated with programmed cell death has provided a prognostic prediction system that has included many genes associated with autophagy, apoptosis, and ferroptosis, but also five genes associated with oxeiptosis. For future research, the authors recommend including newly discovered genes, such as oxeiptosis-associated genes, to increase the scope of gene panel analyses and improve the predictive ability of these analyses [37].

Entosis as a general term refers to the invasion of a living cell into another cell of the same type, forming a “cell-in-cell” (CIC) structure. This process depends on the accumulation of actinomycin at the cell surface as well as E-cadherin, Ras homolog family member A (RHOA), actin, and ezrin [38]. Later, the engulfed cells are eliminated by autophagy-related and caspase-independent cell death involving autophagy-related proteins such as ATG5 and ATG7 [38,39]. Entotic cell death occurs in various types of cancer and, depending on the type of cancer, entosis may have a tumor-promoting effect or be associated with a more favorable outcome for the patients. A correlation between the occurrence of entosis and a poorer prognosis for patients has been demonstrated for squamous cell carcinomas of the head and neck and for pancreatic carcinomas, while a correlation with a better prognosis has been shown for rectal carcinomas. For breast cancer, in particular, the question of a correlation between entosis and a worse or better prognosis for the patient depends on the histological type of carcinoma [38,187]. No experience has yet been reported on the phenomenon of entosis in gliomas. Panoptosis is defined as a form of cell death in which pyroptosis, apoptosis, and necroptosis are activated in the same cell population [40]. It was discovered as a phenomenon that occurs after pharmacological induction or through exposure to pathogens such as the influenza virus type A. Panoptosis leads to the simultaneous activation of key apoptosis molecules such as caspase 8 and caspase 9, key molecules of pyroptosis such as caspase 1 and members of the pyrin domain of the NLR family (NLRP), and key molecules of necroptosis such as receptor-interacting protein kinase 1 and 3 (RIPK 1, RIPK 3), and mixed lineage kinase domain-like (MLKL) [40,41]. Two other key regulators of panoptosis are Z-DNA-binding protein 1 (ZBP1) and transforming growth factor ß-activated kinase 1 (TAK1). ZBP1 contains a RIP domain with a homotypic interaction motif (RHIM domain) that facilitates the activation of various molecules associated with cell death and panoptosis in general, while TAK1, which is typically involved in pro-survival signaling, leads to panoptosis when inactivated [41]. An experimental study on panoptosis induced by bacterial and viral triggers showed that the combined deletion of panoptotic proteins in knock-out mice protected macrophages from cell death, while deletion of individual proteins provided reduced or no protection against panoptosis. Co-immunoprecipitation of panoptotic proteins supports the view that they are assembled in a single molecular complex, the “panoptosome” [42]. The occurrence of panoptosis in tumors has been confirmed by panoptosis-related gene expression analyses and by mutation analyses in cell lines of different tumor types such as renal cell carcinoma, skin melanoma, and glioma [188,189]. In gliomas, a higher frequency of driver gene mutations with high expression of panoptosis-related genes has been confirmed [188]. The biological significance of two panoptosis-related predictors, MYB proto-oncogene like 2 (MYBL2) and tubulin alpha-1C chain (TUBA1C), was also confirmed by the experimental knockdown of both predictors, which resulted in a significant inhibition of glioma cell proliferation and migration. These two predictors belong to a group of panoptosis-related genes that show a significant correlation with TEM in gliomas, and a low score for these genes correlates with the degree of anti-tumor immune cell infiltration in TEM. However, the authors point out that the mechanism of panoptosis has not yet been fully elucidated and that validation of the function of various panoptosis-related genes should be considered an important direction for glioma research [189].

The complete overview of the forms of programmed cell death (PCDs) described to date confirms the realistic future prospect of therapeutically influencing selected molecular components of their signaling pathways. The two principal ways are the activation of individual or several PCDs in tumor cells to achieve a tumor-suppressive effect or blocking the cell death of immunocompetent immune cells in order to prevent an immunosuppressive and thus tumor-promoting constellation of the components of TEM. A particular challenge here is the ambivalent effect of many PCDs with regard to a tumor-promoting or tumor-inhibiting effect, which can be observed in many tumor types such as gliomas [16,37,79,90]. PCDs such as NETosis, oxeiptosis, entosis and panoptosis, which have not yet been investigated in gliomas or only to a limited extent, should also be considered in the development of therapeutic concepts in the future, with panoptosis in particular representing a promising model for the simultaneous targeting of different forms of PCDs [188]. At present, efforts to therapeutically influence the molecular components of PCDs in gliomas have been limited to in vitro studies and experimental in vivo studies. A review of the application of natural small-molecular compounds as targets for programmed cell death in gliomas has shown that the induction of programmed cell death by this substance group is indeed possible. Small-molecular compounds are organic compounds with molecular weights of less than 900 Da. Their application to glioma cells represents a major advancement, as the morphological and clinical success of molecular treatment of gliomas has so far been limited. It has been shown that ferroptosis in glioma cells can be induced by the small-molecular compound pseudolaric acid B (PAB), a diterpenoid acid with known anti-tumor capacity. An induction of necroptosis in glioma cells by dehydrobastidine, a compound from the argentinean mugwort plant, Artemisia douglasiana Besser, was also achieved [190]. In our own literature research, we have focused primarily on therapeutic approaches since 2021. With this research, we can highlight an unbroken interest in the therapeutic targeting of non-apoptotic PCDs in gliomas, regardless of the type of substance class or the type of application of the compound used. The results of representative studies of the last three years have already shown that the therapeutic manipulation of PCDs represents a clear perspective for future glioma research, which should be pursued further. An important example of recent studies is the effect of cannabidiol (CBD), which induces mitochondrial dysfunction in vitro and in vivo, leading to autophagic cell death of glioma cells. The calcium ion channel TRPV4 (“transient receptor potential cation channel subfamily V member 4”) was discovered as a major factor involved in the growth inhibitory effect of CBD. In addition, the combination therapy of CBD and temozolomide resulted in a synergistic effect in terms of tumor growth inhibition and survival time in a mouse model [181]. The thrombin inhibitor hirudin has shown significant antitumor activity in various cancers, but the exact mechanism is still unclear. In glioma cells, hirudin led to autophagic cell death mediated by the inhibition (phosphorylation) of the mTOR pathway and its downstream substrates ULK (“Unc-51 like kinase complex”), P70S6K (“ribosomal protein S6 kinase beta-1”), and 4EBP1 (“eukaryotic translation initiation factor 4E-binding protein 1”). Additionally, hirudin inhibited the proliferation, migration, and invasion of glioma cells in vitro and in vivo in xenotransplanted mice [191]. Menadione (2-methyl-1,4-naphthoquinone) is a fat-soluble synthetic analog of vitamin K known for its anticancer properties due to antiproliferative and cytotoxic effects leading to caspase-dependent and caspase-independent tumor cell death in various cancer cell lines. The antioxidant ascorbic acid is able to enhance the cytotoxic effect of menadione by increasing menadione-induced ROS accumulation. The combined application of menadione and ascorbic acid in a glioblastoma cell line induced autophagic cell death of glioma cells, accompanied by an upregulation of autophagy markers such as LC3, Beclin1 and the ULK complex. This treatment led to a strong reduction in the viability of glioma cells. Therefore, the combination of menadione and ascorbic acid is considered a potential approach for glioblastoma therapy [192]. Fatostatin is a specific inhibitor of sterol regulatory element binding proteins (SREBPs), which is involved in lipid and cholesterol synthesis and has an antitumor effect in various tumors. It induces ferroptosis in glioblastomas by inhibiting the AKT/mTOR/4EBP1 axis, and administration of fatostatin by nanoparticles resulted in the inhibition of glioma growth in an intracranial xenograft mouse model [182]. Gastrodin, a compound isolated from the orchid Gastrodia elata, is another compound capable of inducing ferroptosis in glioma cells by enhancing its main target homeobox 10 (HOXD10). In a subcutaneous mouse model, gastrodin led to enhanced expression of HOXD10, induced ferroptosis of glioma cells, and suppressed glioma growth in vivo [193]. Inspired by the boron neutron capture therapy, a new form of cancer therapy that is currently being researched using boron-containing agents and ionizing radiation [194], borax (sodium borate) induced ferroptosis in glioma cells in vitro by inhibiting the HSPA5/NRF2/GPX4/GSH signaling pathways. This led to a significant suppression of tumor cell viability and proliferation in various glioma cell lines [183]. Azole compounds are known for their antibacterial, antifungal, and antitumor potential. The three azole compounds flubendazole, mebendazole, and fenbendazole inhibited the proliferation and migration of glioma cells in various cell lines and led to pyroptosis of tumor cells via the activation of the nuclear factor kappa-light-chain-enhancer of activated B-cell (NF-kB) complex. Even in a xenograft mouse model, flubendazole suppressed tumor growth with disappearance of tumors after 24 days and without observable side effects in vivo [195]. Pyroptosis of glioma cells was also induced by a live attenuated oncolytic vaccine strain of Zika virus (ZIKV-LAV), which was confirmed by increased cellular secretion of interleukin-1ß (Il-1ß) in virus-infected glioma cells. The induction of cell death was restricted to the glioma cells and did not occur in terminally differentiated neurons or endothelia (Table 2) [196].

In conclusion, the presentation of the current state of knowledge on the non-apoptotic forms of programmed cell death (PCD) in gliomas shows that numerous and diverse insights have already been gained into the molecular signaling pathways of these PCDs and their influence on the biology of tumors. One of the most important findings is the fact that this influence on tumor biology differs between different types of tumors. Many forms of PCDs tend to have a tumor-promoting effect in some tumor types, while in others, they tend to contribute to the suppression of tumor growth. It was therefore important to focus the present review of PCDs on one tumor type, namely gliomas. Another important finding is the proof of the correlation of the expression of genes related to most forms of non-apoptotic cell death with a poorer prognosis and shorter survival time. In addition, many research groups have shared the assumption that this correlation can be explained, at least in part, by increased cell death of the immunocompetent cells of the tumor environment (TEM), which leads to immunosuppression with a tumor-promoting effect. In this context, the resistance of immunosuppressive cells to individual types of PCDs also plays a role. In contrast, many experimental studies have shown that the spontaneous induction of non-apoptotic PCDs predominantly has a tumor suppressive effect, confirming the contrasting effect of PCD on glioma biology. Research into the exact role of non-apoptotic forms of PCD in gliomas is still at an early stage. The main perspectives for the future are to gain further insight into the molecular interaction between the PCD forms, to gain more insight into the recently discovered forms of programmed cell death such as cuproptosis and panoptosis, and to continue the already promising approaches for molecular targeting as a prerequisite for therapeutic studies on humans. 

## Figures and Tables

**Table 1:** Overview of all forms of non-apoptotic cell death discussed in the present review.

**Autophagy and autophagic cell death:** The self-preservation of a cell through the degradation of its own cellular components under physiological conditions is called “autophagy”. The term “autophagic cell death” is limited to forms of cell death associated with overactivation of the autophagic molecular cascade [7,8,9].
**Ferroptosis:** Originally described as “a unique iron-dependent form of non-apoptotic cell death”, it can be triggered by various molecular pathways such as the “Fenton reaction”. Ferroptosis shows different effects in tumor biology, from inhibition to promotion of tumor progression [11,12].
**Pyroptosis:** A form of cell death so named because it was first described in macrophages after a Salmonella infection. Pyroptosis plays a role in various forms of infections, but also in other diseases such as cancer [13,14,15].
**Necroptosis:** The name is based on the morphological similarity to necrosis. However, necroptosis is defined by a specific and caspase-independent molecular cascade that clearly distinguishes necroptosis from other forms of cell death [16,17,18,19].
**Cuproptosis:** A disturbance of copper homeostasis can lead to copper overload with subsequent induction of this form of cell death. The role of cuproptosis in neurodegenerative diseases and various types of cancer has already been confirmed [20,21,22,23].
**Paraptosis:** A form of cell death that does not respond to caspase inhibitors. In contrast to many other forms of cell death, which have different effects in tumor biology, paraptosis is predominantly thought to have a tumor-inhibiting effect [24,25,26].
**Methuosis:** Macropinocytosis activated by a specific molecular cascade is the main feature of methuosis, which is a predominantly tumor-suppressive form of cell death. A growing number of compounds are currently being described, all of which can trigger the phenomenon of methuotic cell death [27,28,29].
**Parthanatos:** A form of cell death that largely depends on an excess of the nuclear enzyme poly-ADP-ribose polymerase-1 (PARP-1). This enzyme can have different cellular effects, ranging from its role in cellular survival to cell death [30,31].
**Anoikis:** Cell death triggered by the disruption of interactions between neighboring cells and the extracellular matrix. The molecular processes induced by this disruption are identical to the extrinsic and intrinsic pathways of apoptosis [32,33].
**NETosis:** The phenomenon of NETosis is triggered by neutrophils forming reticular structures called neutrophil extracellular traps (NETs), which consist of various nuclear and cytoplasmic components. It can be triggered by various stimuli such as immune complexes, cytokines, and chemokines, and can lead to caspase-independent cell death [34,35].
**Oxeiptosis:** A caspase-independent form of cell death triggered by reactive oxygen species (ROS) and a specific molecular cascade, leading to the dephosphorylation of apoptosis-inducing mitochondria-associated factor 1 (AIFM1) at site S116 [36,37].
**Entosis:** The invasion of a living cell into another cell of the same type, forming a “cell-in-cell” (CIC) structure, is called “entosis”. After cellular invasion, the engulfed cells undergo caspase-independent cell death [38,39].
**Panoptosis:** A phenomenon triggered by various stimuli in which pyroptosis, apoptosis, and necroptosis are initiated in the same cell population following the activation of key molecules from the signaling pathways of all three forms of cell death [40,41,42].

**Table 2:** Important examples of in vitro studies and experimental in vivo studies reported in 2021–2024 targeting key molecules of different forms of non-apoptotic cell death to attenuate the growth of gliomas and glioma cells. Abbreviations in alphabetical order: 4EBP1: eukaryotic translation initiation factor 4E-binding protein 1; AKT: protein kinase B; CBD: cannabidiol; GPX4: glutathione peroxidase 4; GSH: glutathione; HOXD10: homeobox 10; HSPA5: heat shock protein family A member 5; Il-1ß: Interleukin-1ß; in vitro: in vitro study; in vivo: experimental in vivo study; LC3: lipidated light chain 3; mTOR: mammalian target of rapamycin pathway; NRF2: nuclear factor erythroid 2-related factor 2; P70S6K: ribosomal protein S6 kinase beta 1; ROS: reactive oxygen specimen; TRPV4: transient receptor potential cation channel subfamily V member 4; ULK: Unc-51 like kinase complex.

First Author, Year, Type of Study	Applied Compound	Type of the Compound
Huang, 2021, in vitro + in vivo [181]	**Cannabidiol (CBD)**	A major cannabinoid from the cannabis plant
CBD induces mitochondrial dysfunction leading to autophagic cell death in glioma cells, whereby the calcium ion channel protein TRPV4 is involved in the growth-inhibiting effect of CBD. The combination therapy of CBD and temozolomide led to tumor inhibition in a mouse model.		
Ma, 2023, in vitro + in vivo [191]	**Hirudin**	Thrombin inhibitor with antitumor potential
Hirudin leads to autophagic cell death of glioma cells by inhibiting the mTOR signaling pathway and its downstream substrates ULK, P70S6K and 4EBP1. Hirudin inhibits proliferation, migration and invasion of glioma cells in vitro and in xenotransplanted mice.		
Despotovic, 2022, in vitro [192]	**Menadione combined** **with ascorbic acid**	Vitamin K analogue combined with an antioxidant
The antioxidant ascorbic acid enhances the cytotoxic effect of menadione by increasing menadione-induced ROS accumulation. The application of menadione in combination with ascorbic acid induced autophagic cell death of glioma cells, accompanied by an upregulation of the autophagy markers LC3, Beclin1 and the ULK complex.		
Cai, 2023, in vivo + in vitro [182]	**Fatostatin**	Regulator of lipid and cholesterol synthesis
Fatostatin induces ferroptosis in glioblastomas by inhibiting the AKT/mTOR/4EBP1 axis. Administration of fatostatin in the form of nanoparticles resulted in inhibition of glioma growth in an intracranial xenograft mouse model.		
Cao, 2023, in vivo + in vitro [193]	**Gastrodin**	Compound from the orchidgastrodia elata
Gastrodin induces ferroptosis in glioma cells by increasing the expression of its main target HOXD10, thereby suppressing the colony and spheroid formation of tumor cells. Gastrodin also inhibits the growth of glioma cells in a xenografted mouse model.		
Tuncer, 2024, in vitro [183]	**Borax**	Sodium borate
Borax induces ferroptosis in glioma cells by inhibiting the HSPA5/NRF2/GPX4/GSH signaling pathways, resulting in significant suppression of tumor cell viability and proliferation.		
Ren, 2021, in vitro + in vivo [195]	**flubendazole, mebendazole, fenbendazole**	Azole compounds with antibacterial, antifungal and antitumor potential
These azole compounds induce pyroptosis of glioma cells and inhibit their proliferation, invasion and cell migration. Flubendazole suppresses tumor growth in a xenograft mouse model with no apparent side effects, and the tumors disappeared within 24 days.		
Victorio, 2024, in vitro [196]	**ZIKV-LAV**	Live attenuated oncolytic vaccine strain of Zika virus
ZIKV-LAV induces pyroptosis of glioma cells after infection. Virus-infected tumor cells showed increased cellular secretion of interleukin-1ß (Il-1ß). The induction of pyroptotic cell death was restricted to the glioma cells and did not occur in terminally differentiated neurons or endothelia.		

## Data Availability

Not applicable.

## Abbreviations

1-NP: 1-nitropyrene; 4EBP1: eukaryotic translation initiation factor 4E-binding protein 1; 5-HT: 5-hydroxytryptamine; αHG: α-hydroxyglutarate; ACSL4: acyl-CoA synthetase long-chain family member 4; ADP: adenosine diphosphate; AIF: apoptosis-inducing factor; AIFM1: mitochondria-associated factor 1; AIP1: ALG-2-interacting protein 1; AKT: protein kinase B; ANT1: adenine nucleotide translocase 1; APAF1: apoptotic peptidase activating factor 1; Arf6: ADP ribosylation factor 6; ATGs: autophagy-related genes; ATP7A/B: Cu-transporting ATPases ATP7A and ATP7B; AVß3: alpha-v beta-integrin; BAF: T-butyloxycarbonyl-Asp-(O-methyl)-fluoromethyl ketone; BAK: BCL2-homologous antagonist/killer; BAX: BCL2-associated X protein; BBB: blood-brain barrier; BCL2: B-cell lymphoma 2 protein; BDNF: brain-derived neurotrophic factor; cAMP: cyclic adenosine monophosphate; CBD: Cannabidiol; CD: cluster of differentiation; CDK1/2: cyclin-dependent kinase 1 and 2; cIAP: cellular apoptosis inhibitor protein; CIC: cell-in-cell structure; circRNAs: circular RNA molecules; CK1: casein kinase 1; COPZ1: coatamer-protein complex subunit zeta 1; COX17: copper chaperone of cytochrome c oxidase; CP: ceruloplasmin; CREB: cAMP response element-binding protein; CRGs: cuproptosis-related genes; Cu: cuprum (copper); CYLD: cylindromatosis tumor-suppressor protein; Cys: cysteine; DAMPs: damage-associated molecular patterns; DLAT: dihydrolipoamide S-acetyltransferase; DPP4: dipeptidyl peptidase 4; DPT: deoxy-podophyllotoxin; EAA1: early endosomal antigen 1; eEF2: elongation factor-2 kinase; EGF: epidermal growth factor; EGFR: epidermal growth factor receptor; ELANE: neutrophil elastase; ER: endoplasmic reticulum; EREG: epiregulin; FDX1: ferredoxin 1; Fe: ferrum (iron); Fe-S cluster: iron-sulphur cluster; FSP1: ferroptosis suppressor protein 1; GABA: gamma-aminobutyric acid; GPX4: glutathione peroxidase 4; GSDMA-GSDME: gasdermins A-E; GSH: glutathione; HCC: hepatocellular carcinoma; HMOX1: heme oxygenase 1; HOXD10: homeobox 10; HSPA8: heat shock protein family A member 6; HSPB1: heat shock protein beta 1; IDH: isocitrate dehydrogenase; IFN-γ: Interferon-γ; IGF2BP3: insulin-like growth factor 2 mRNA-binding protein 3; IGFIR: human insulin-like growth factor 1 receptor; Il-6: interleukin 6; iPA: N6-isopentenyladenosine; JNK: Jun-N-terminal kinase 1; KEAP1: kelch-like enoyl-CoA hydratase-associated protein 1; KFERQ-like motif: a peptide sequence consisting of lysine (K), phenylalanine (F), glutamic acid (E), arginine (R), and glutamine (Q); KLF9: krüppel-like factor 9; KRASG12D: mutation of the oncogene kirsten rat sarcoma virus with exchange of the amino acid glycine (G) by aspartic acid (D) at position 12; LAMP1: lysosome-associated membrane protein 1; LC3: lipidated light chain 3; lncRNAs: long non-coding RNA molecules; MAPK: mitogen activated protein kinase pathway; MDA9: melanoma differentiation associated gene-9/syntenin; MDSCs: myeloid-derived suppressor cells; MEK2: mitogen-activated protein kinase 2; MEKK-1: mitogen-activated protein kinase kinase-1; MIPP: methyl-1H-indole-pyridinyl-propene-1; miRNAs: micro RNA molecules; MLKL: mixed lineage kinase domain-like; MMP2: metalloprotease 2; MNX1: motor neuron and pancreatic homeobox 1; MOMIPP: methoxy-methyl-1H-indole-pyridinyl-propene-1; mTOR: mammalian target of rapamycin pathway (mTOR); MYBL2: MYB proto-oncogene like 2; NC: nucleolin; ncRNAs: non-coding RNA molecules; NF-kB: nuclear factor kappa-light-chain-enhancer of activated B-cell; NGF: nerve growth factor; NK1R: neurokinin-1 receptor; NLRC4: NLR family CARD domain containing 4; NLRP2: NLR family pyrin domain containing 2; NMDA: N-methyl-D-aspartate receptor; nNOS: neuronal nitrite oxide synthase; NO: nitrite oxide; NOD1: nucleotide-binding oligomerization domain-containing protein 1; NOX: NADPH oxidase complex; NRGs: necroptosis-related genes; ODF3B: outer dense fibers of sperm tail 3B; P70S6K: Ribosomal protein S6 kinase beta-1; PAK4: P21-activated kinase 4; PAMPs: pathogen-associated molecular patterns; PAR: poly-ADP-ribose; PARP-1: poly-ADP-ribose polymerase-1; PCD: programmed cell death; PDGFR: platelet-derived growth factor receptor; PD-L1: programmed cell death ligand 1; PDZK1: PDZ domain-containing protein 1; PGAM5: phosphoglycerate mutase 5; PIK3C3: phosphatidylinositol 3-kinase complex with type 3 catalytic subunit; PJVK: Pejvakin; PKC: protein-kinase C pathway; PRGs: pyroptosis-related genes; PUFAs: polyunsaturated fatty acids; RAB7: Ras-related protein member 7; Rac1: Rac family small GTPase 1; RAS: proto-oncogene ‘rat sarcoma’; RDG-sequence: arginine-aspartic acid-glycine sequence; RHIM: homotypic interaction motif; RHOA: Ras homolog family member A; RIPK1/3: receptor-interacting protein kinase 1 and 3; ROS: reactive oxygen species; SIRT1: sirtuin-1; SLC31A1: solute carrier family 31 member 1; SLC40A1: solute carrier family 40 member 1; SLC7A11: solute carrier family 7 member 11; SLC9A1: solute carrier family 9 member 1; SOD1: Cu-zinc superoxide dismutase 1: SREBPs: sterol regulatory element binding proteins; T3SS: salmonella-specific islet-1 type III secretion system; TAC-cycle: tricarboxylic acid cycle; TAK1: transforming growth factor ß-activated kinase 1; TAX1BP1: Tax 1-binding protein 1; TEM: tumor environment; TLR4: toll-like receptor 4; TMEM164: transmembrane protein 164; TMZ: temozolomide; TNFR1: tumor necrosis factor receptor 1; TNF-α: tumor necrosis factor α; TP53: tumor protein 53; TRAIL: tumor necrosis factor-related apoptosis-inducing ligand; Tregs: T-regulatory cells; TrkB: tyrosine kinase receptor B; TRPV4: transient receptor potential cation channel subfamily V member 4; TUBA1C: tubulin alpha-1C chain; UBE2C: ubiquitin-conjugating enzyme E2C; ULK: Unc-51-like kinase complex; VPS34: vacuolar protein sorting 34; VR1: vanilloid receptor subtype 1; WNT: wingless-type signaling pathway; Xc-: cystine/glutamate antiporter system Xc-; ZBP1: Z-DNA binding protein 1; ZIKV-LAV: live attenuated oncolytic vaccine strain of Zika virus.
